# ABA signaling components in *Phelipanche aegyptiaca*

**DOI:** 10.1038/s41598-019-42976-3

**Published:** 2019-04-24

**Authors:** Gil Wiseglass, Oded Pri-Tal, Assaf Mosquna

**Affiliations:** The Robert H. Smith Institute of Plant Sciences and Genetics in Agriculture, the Hebrew University of Jerusalem, Rehovot, 7610001 Israel

**Keywords:** Plant hormones, Plant evolution

## Abstract

Obligate root holoparasite *Phelipanche aegyptiaca* is an agricultural pest, which infests its hosts and feeds on the sap, subsequently damaging crop yield and quality. Its notoriously viable seed bank may serve as an ideal pest control target. The phytohormone abscisic acid (ABA) was shown to regulate *P*. *aegyptiaca* seed dormancy following strigolactones germination stimulus. Transcription analysis of signaling components revealed five ABA receptors and two co-receptors (PP2C). Transcription of lower ABA-affinity subfamily III receptors was absent in all tested stages of *P*. *aegyptiaca* development and parasitism stages. *P*. *aegyptiaca* ABA receptors interacted with the PP2Cs, and inhibited their activity in an ABA-dependent manner. Moreover, sequence analysis revealed multiple alleles in two *P*. *aegyptiaca* ABA receptors, with many non-synonymous mutations. Functional analysis of selected receptor alleles identified a variant with substantially decreased inhibitory effect of PP2Cs activity *in-vitro*. These results provide evidence that *P*. *aegyptiaca* is capable of biochemically perceiving ABA. In light of the possible involvement of ABA in parasitic activities, the discovery of active ABA receptors and PP2Cs could provide a new biochemical target for the agricultural management of *P*. *aegyptiaca*. Furthermore, the potential genetic loss of subfamily III receptors in this species, could position *P*. *aegyptiaca* as a valuable model in the ABA perception research field.

## Introduction

The obligate root holoparasite weed *Phelipanche aegyptiaca* (Egyptian broomrape), is a species from the Orobanchaceae family, which includes some of the most agriculturally damaging weeds^1–3^. *P*. *aegyptiaca* is harmful, especially owing to its ability to parasitize a large variety of crop families, including *Solanaceae*, *Brassicaceae*, *Cucurbitaceae*, *Cruciferae*, *Apiaceae*, *Fabaceae* and *Asteraceae*^4^. As an obligate holoparasite lacking chlorophyll and effective roots, *P*. *aegyptiaca* depends entirely on its host for nutrients and water^5^. Germination of *P*. *aegyptiaca* and other obligate Orobanchaceae parasites requires strigolactones, stimuli derived from the host plant^6^. Exposure of conditioned *P*. *aegyptiaca* and *P*. *ramose* seeds to a synthetic germination stimulant similar in structure to strigolactones, was rapidly followed by a considerable elevation in CYP707A1 transcript levels and reduction of seed abscisic acid (ABA) content^7–9^. Cytochrome P450 CYP707A encodes ABA 8′-hydroxylase, a key enzyme in ABA catabolism, and plays a role in relieving seed dormancy^10^. Thus, an antagonistic relationship between strigolactones and the germination inhibitor ABA stand at the basis of germination regulation in parasitic Orobanchaceae species.

ABA elicits its effect by binding pyrabactin resistance1/PYR1-like/regulatory component of ABA receptor (PYR/PYL/RCAR) ABA receptors in a large and conserved hydrophobic pocket, which changes the conformation of two highly conserved loops located in the outer periphery of the pocket^11–13^. Both loops, the “gate” and the “latch”, move towards the ligand and in doing so “cover” the pocket cavity^13^. This structural shift enables the occupation of the catalytic core of protein phosphatases type 2CA (PP2CA), the ABA co-receptors, in a manner which blocks its activity^13^. *Arabidopsis thaliana* PP2CA ABI1 and ABI2 were the first confirmed negative regulators of ABA signaling^14^, where the ABA-mediated interaction between PYR/PYLs and ABI1/ABI2 was shown to inhibit the phosphatase activity *in-vitro* and to antagonize their action *in-planta*^11,12^.

Many of the ABA receptor functions can be attributed to their sensitivity to ABA and affinity to PP2C, i.e., the concentration of ABA which elicits a receptor-PP2C interaction. Dimeric ABA receptors have been shown to require higher concentrations of ABA to elicit the same activity as monomeric receptors^15^. In *Arabidopsis*, deficiency in three dimeric receptors was associated with measurable ABA insensitivity and a reduced inhibitory effect of ABA on seed germination^12^.

Autotroph plants utilize ABA as an inductive signal in a wide range of responses and physiological functions vital to their survival and reproduction. Few of these functions, e.g., stress-related responses in the roots and leaves, have not been identified in obligate holoparasitic plants, such as *P*. *aegyptiaca* that rely completely on their host for continuous supply of water and nutrients. The reduction in ABA-related functions in holoparasitic plants might correspond with some degree of degeneration in the ABA signal transduction pathway. This hypothesis is strengthened by the recent identification and classification of ABA receptors and co-receptors in the hemiparasitic Orobanchaceae species *Striga hermonthica*, which transcribes four ABA co-receptors, including one which is mutated in such way that it effectively blocks ABA signaling^9^.

In this study, we explored ABA perception in *P*. *aegyptiaca* and provide early insights into genetic variance and its functionality in a wild species. Alongside the potential evolutionary implications of such discoveries, the insights may also illuminate new approaches for agrotechnical control of this pest.

## Results

### *P*. *aegyptiaca* transcribes core ABA signaling components

The basis of the biochemical response to ABA is facilitated by an interaction between ABA and its receptor, followed by the ABA-receptor inhibitory effect on a co-receptor (PP2CA). Identification of these components in *P*. *aegyptiaca* was based on sequence homology with *Arabidopsis* ABA receptor PYR1 and ABA co-receptor ABI1. In the absence of a publically available sequenced genome, the Parasitic Plant Genome Project (PPGP) EST database is currently the most extensive source of information about *P*. *aegyptiaca* genetics. The database contains cDNA sequences obtained from *P*. *aegyptiaca* and two other parasitic plant species at specific developmental stages and from different tissues^16^.

**Figure 1:**
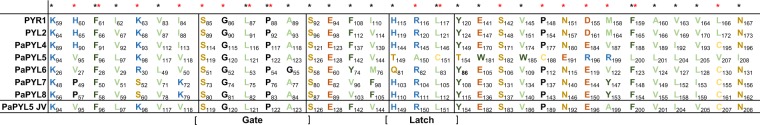
High sequence similarity between the ABA receptors of *P*. *aegyptiaca* and of *A*. *thaliana* in regions key to functionality. Alignment of residues which interact with ABA (black asterisks) or HAB1 (red asterisks), according to the crystal structures of PYL2-ABA^13^ and PYR1-HAB1^17^. Amino acid sequences are color-coded according to side chain characteristics. PaPYL5 JV is the variant which was amplified from a sample obtained in the Jezreel Valley (Israel).

*In-silico* analysis of *P*. *aegyptiaca* transcript data revealed five putative ABA receptors (PaPYL4-8) and five putative ABA PP2C co-receptors (Figs 1 and S1). Regions predicted to be key to receptor functionality^13,17^ in the amino acid sequences of the putative ABA receptors were found to be highly similar to those of the *Arabidopsis* ABA receptors (Fig. 1). One exception was PaPYL5, which varied from both *Arabidopsis* and the rest of the *P*. *aegyptiaca* receptors in a highly conserved region, which includes the “latch” loop (PYR1 H_115_, R_116_ and L_117_) (Fig. 1). The latch is one of two surface loops that bind ABA and co-receptors. A change in this region may therefore affect ABA receptor function.

**Figure 2:**
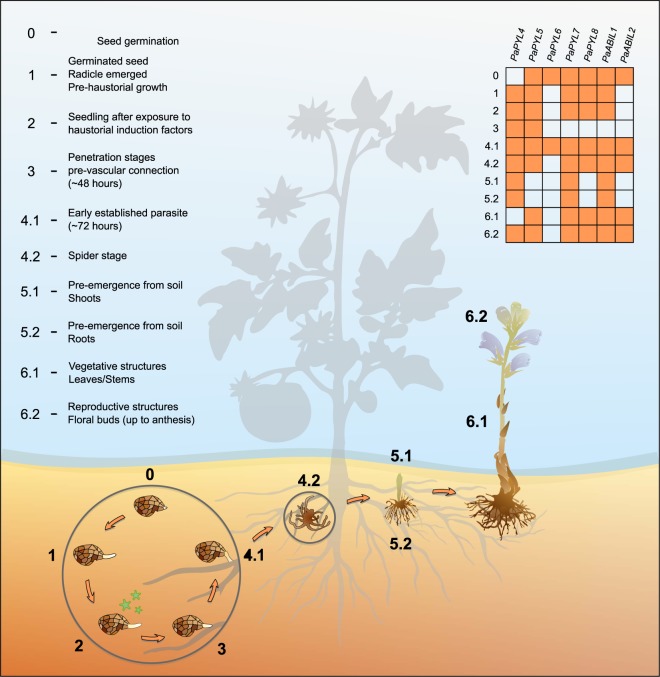
*Orobanche* sp. life cycle and ABA receptors and co-receptors transcription patterns. (**0–4**) Seed germination is stimulated by host-derived strigolactones. Seedlings grow in a chemotropic manner, towards increasing concentrations of the stimulant, and establish cellular connections with the host xylem and phloem. **(5)** The young parasite continues to develop underground until it is ready to reproduce; then, the flowering shoots emerge from the surface. **(6)** Each flower can produce around 500 seeds by cross-pollination, self-pollination and apomixis. In table: orange boxes represent highly similar (>95% nucleotide identity in pairwise sequence alignment) matches in the Parasitic Plants Genome Project database. To eliminate the possibility of the matches that were the product of a contaminated genetic material (host tissues), the uniqueness of each match was verified by basic local alignment search (BLAST) in the NCBI database.

The individual transcription pattern of the putative ABA receptors and co-receptors was analyzed using the publically available PPGP transcriptome data, which are categorized by developmental stage and tissue type (summarized on Fig. 2). Results showed that at least two ABA receptors are transcribed at any given stage of *P*. *aegyptiaca* life cycle, which can be an indication of active ABA perception. *PaPYL6* and the *ABI-like 2* co-receptor (*PaABIL2*) were only transcribed during seed germination and early established parasite stage. *PaABIL2* was also transcribed during “post-emergence from soil” stage.

### None of the transcribed *P*. *aegyptiaca* ABA receptors classify as a subfamily III ABA receptor

**Figure 3:**
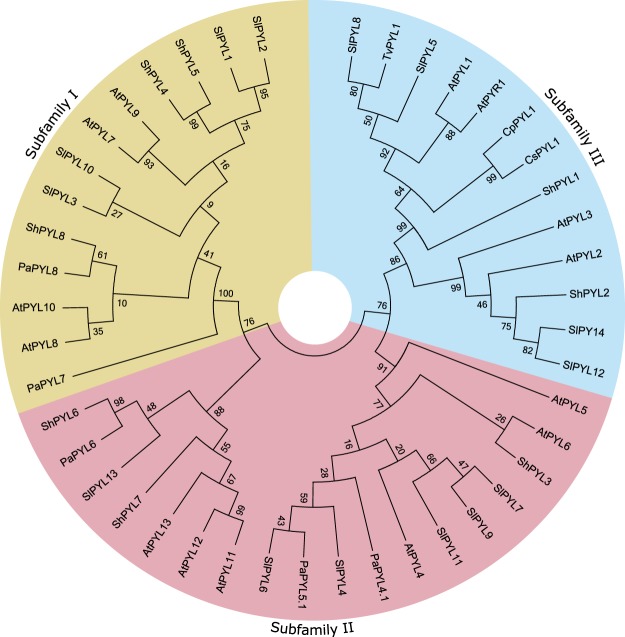
Phylogenetic-based subfamily classification of ABA receptors. A th*aliana* (AtPYL)^11,12^, tomato (SlPYL)^18,33^ *Striga hermonthica* (ShPYL)^9^ *P*. *aegyptiaca* (PaPYL) and selected putative ABA receptors of parasitic plant species (CpPYL-*C*. *pentagona*, TvPYL*-T*. *versicolor*, *CsPYL-C*. *suaveolens)*. See methods for phylogenetic analyses experimental procedures.

*In-silico* phylogenetic analysis clustered PaPYL4-6 with subfamily II of *A*. *thaliana* ABA receptors, and PaPYL7 and 8 with subfamily I (Fig. 3). None of the putative *P*. *aegyptiaca* ABA receptors clustered with subfamily III, an unusual finding as compared to ABA receptor expression analyses in other higher plants^9,18–23^. Thus we decided to number the receptors in accordance to *Arabidopsis* subfamily clustering. Subfamily II receptors were named PaPYL4, 5 and 6 and Subfamily I receptors were named PaPYL7 and PaPYL8.

A functional analysis was then performed to confirm the computational phylogenetic classification of putative *P*. *aegyptiaca* ABA receptors into subfamilies I and II. To this end, the five receptors were cloned from plant samples collected in Israel. The cloned receptors were highly similar to the PPGP database sequences, with the exception of PaPYL5. The latch loop of this variant, unlike its PPGP counterpart, was found to be conserved as compared to other functional receptors. This version was named PaPYL5 JV after the source of the sample - Jezreel Valley.

**Figure 4:**
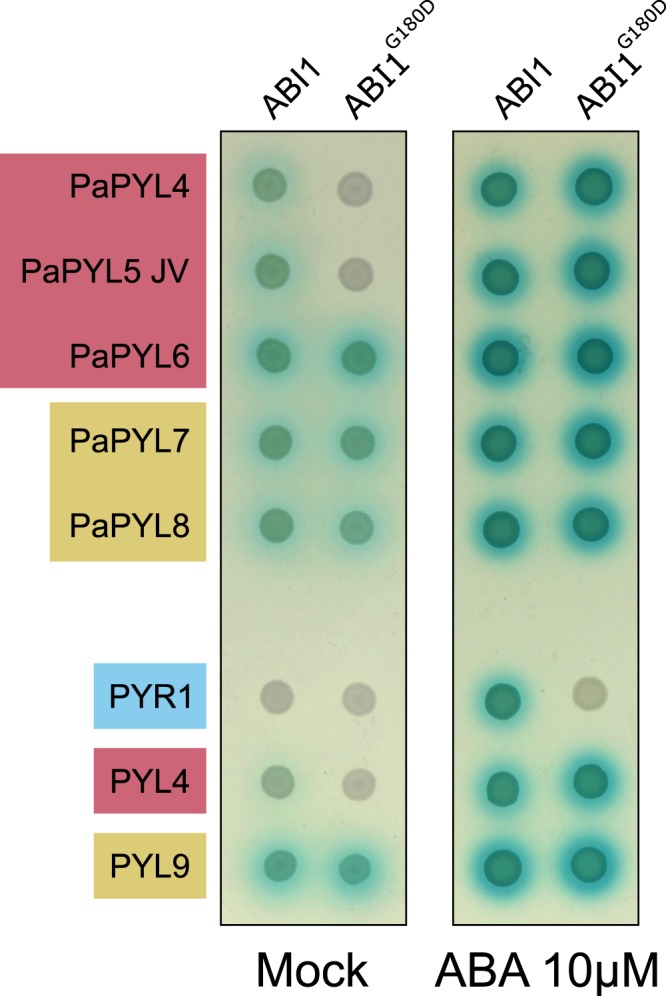
Classification of PaPYL4–8 subfamilies. A yeast two-hybrid interaction assay was performed between putative *P*. *aegyptiaca* ABA receptors fused to a binding domain and *A*. *thaliana* ABA co-receptor ABI1 and its mutant ABI1^G180D^ fused to an activating domain, in the presence of Mock (0.1% DMSO) (left) or 10 µM ABA (right). The models of the defining differences between the ABA receptor subfamilies are represented by PYR1 (subfamily III), PYL4 (subfamily II) and PYL9 (subfamily I), each fused to a binding domain. See methods for phylogenetic analyses experimental procedures.

The interactions between ABA receptors and *A*. *thaliana* ABA co-receptors ABI1 and its mutant ABI1^G180D^ (encoded by *abi1-1*) in a yeast two-hybrid assay, can be used as an indication of a subfamily affiliation^22^. PaPYL4 and PaPYL5 interacted with ABI1 in an ABA-independent manner, while the interaction with ABI1^G180D^ was ABA-dependent (Fig. 4), which coincided with the characteristics of the *A*. *thaliana* ABA receptor subfamily II. PaPYL6-8 interacted with both ABI1 and ABI1^G180D^ in an ABA-independent manner, in accordance with the characteristics of the *A*. *thaliana* ABA receptor subfamily I.

In order to determine whether the absence of subfamily III transcription is a common feature of parasitic plants, sequences encoding putative ABA receptors of the following species were analyzed *in-silico*: obligate root hemiparasite *Striga hermonthica*^9^, facultative root hemiparasite *Triphysaria versicolor* (*Orobanchaceae*, EST libraries available in the PPGP website) and obligate stem holoparasites *Cuscuta pentagona* and *Cuscuta suaveolens*^24^ (EST libraries available in the GenBank TSA database). In all tested species, unlike in *P*. *aegyptiaca*, at least one putative ABA receptor clustered with the subfamily III ABA receptor family (Fig. 3).

### *P*. *aegyptiaca* ABA receptors interact with *P*. *aegyptiaca* ABA co-receptors and inhibit their activity in an ABA-*dependent* manner

**Figure 5:**
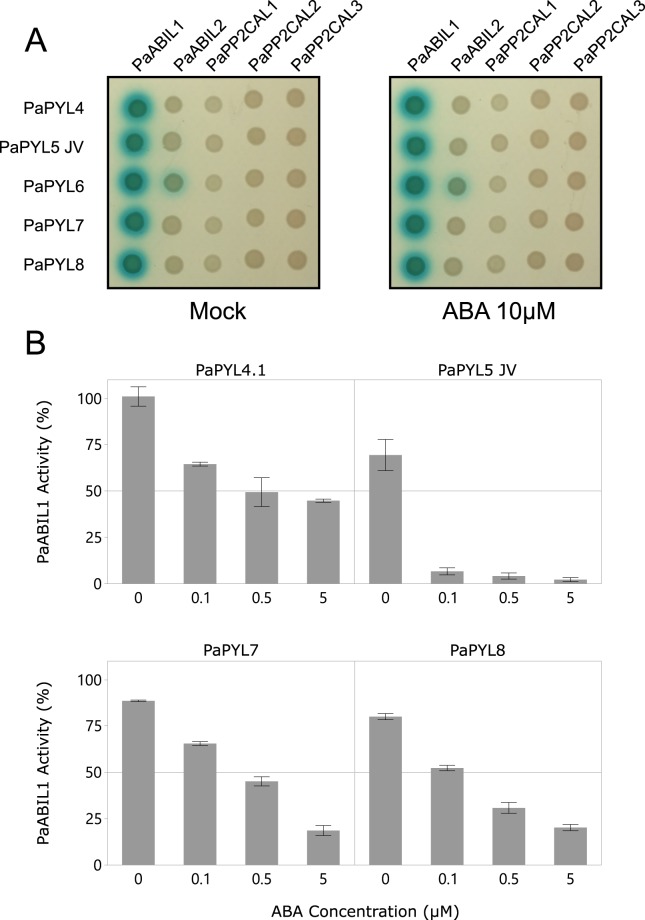
*P*. *aegyptiaca* ABA receptors interact with and inhibit co-receptors. (**A**) Interaction between binding-domain-fused PaPYL4–8 and activating domain-fused PaABIL1 and PaABIL2 in yeast, in the presence of Mock (0.1% DMSO) or 10 µM ABA. (**B**) Recombinant GST-PaABIL1 activity (in %) was measured in the presence of recombinant 6XHIS-SUMO-PaPYL4.1, 6XHIS-SUMO-PaPYL5 or GST-PaPYL7, GST-PaPYL8 and ABA. Each reaction contained a 1:3 molar ratio of PP2C:receptor and was supplemented with increasing concentrations of ABA (0, 0.1, 0.5 or 5 µM). The activity of GST-PaABIL1 was measured for 30 min and phosphatase activity was calculated in two technical replicates. All calculations were performed in the linear phase of the reaction. Values represent percent activity as compared to GST-PaABIL1 tested in the absence of receptors or ABA (Mock). Bars represent a single standard deviation.

Of the five putative ABA co-receptors identified in the *in-silico* analysis of the *P*. *aegyptiaca* transcriptome, only two were shown to interact with *P*. *aegyptiaca* ABA receptors in the yeast two-hybrid assay (Fig. 5). Furthermore, only these two co-receptors interacted with *Arabidopsis* SnRK (Fig. S1). The interaction between *P*. *aegyptiaca* ABI like 1 (PaABIL1) and PaPYL4-8 was ABA-independent. PaABIL2 only interacted with PaPYL6. The other three putative *P*. *aegyptiaca* clade A subfamily of type II C protein phosphatases like 1–3 (PaPP2CAL1-3) ABA co-receptors did not interact with any of the receptors. A receptor-mediated phosphatase activity assay performed to further investigate the interaction between recombinant PaABIL1 and PaPYL4-8 showed that PaPYL4, PaPYL5, PaPYL7 and PaPYL8 inhibited the de-phosphorylation activity of PaABIL1 in an ABA dose-dependent manner (Fig. 5).

### Allelic variations in PaPYL4 affect its interaction with co-receptors

As part of the characterization of *P*. *aegyptiaca* ABA receptors, multiple genes encoding PaPYL4-5 originating from the Jezreel Valley (32° 35′ 47″N, 35°14′31″E region) population, were cloned and sequenced. *In-silico* analysis revealed that PaPYL4 and PaPYL5 presented both synonymous and non-synonymous mutations. Amongst PaPYL4 clones, 35 different alleles were discovered, 6 of which had nucleotide insertions or deletions resulting in a frame-shift. PaPYL5 clones included 12 different alleles, one with a frame-shift and three with nonsense mutations. Assessment of the interaction between the 11 PaPYL4 alleles with complete open reading frames and ABA co-receptors (PaABIL1, PaABIL2, HAB1, ABI1, ABI1^G180D^, ABI2 and ABI2^G168D^) in a yeast two-hybrid assay (Fig. S2), showed that the allelic variation did not affect this interaction, regardless of ABA concentration. However, variant PaPYL4.2 showed a substantially decreased interaction with all the ABA co-receptors, manifested by higher ABA concentration requirements, and failure to interact with the mutated co-receptors at any tested concentration of ABA. In comparison to the normally interacting receptor, PaPYL4.1, PaPYL4.2 displayed no *in-vitro* PP2C inhibition activity, even in the presence of 5 µM of ABA (Fig. S3).

## Discussion

This work presented evidence of the capacity of an obligate holoparasitic plant, *Phelipanche aegyptiaca*, to biochemically perceive ABA signaling, by the apex components of the ABA signal transduction pathway. Through characterization of the plant’s ABA receptors and co-receptors and comparison with homologous autotrophic angiosperm genes, we propose a possible deterioration of the *P*. *aegyptiaca* ABA perception mechanism. This might be the result of the evolutionary transition of this species from self-dependence to parasitism, in which loss of redundancies in once critical traits can occur without decreasing fitness.

The first and most prominent element hinting to a reduction in *P*. *aegyptiaca* ABA perception, was the absence of subfamily III ABA receptor transcription. This was unique as compared to other species of higher plants, which consistently expressed receptors of all three ABA receptor subfamilies^9,18–23^. *Arabidopsis* ABA receptor subfamily III is comprised of dimeric receptors^15,25^, which, recent data suggest, are main mediators of the downstream transcription effect of ABA^26^. Activation of dimeric receptors requires higher levels of ABA in comparison to monomeric receptors, suggestive of an advanced, modular response mechanism. Evidence of subfamily III receptor transcription in *Orobanchaceae* hemiparasitic species and in other holoparasitic plants, suggests that the absence of transcription might be limited to *Orobanche* species, or perhaps only to *P*. *aegyptiaca*. This possibility could be explored pending release of the sequenced genomes of *P*. *aegyptiaca* and other Orobanche species, which will allow us to unequivocally determine whether subfamily III genes are present, lost or merely not transcribed. Loss of the gene expression would coincide with previous evidence of key autotrophic genes which are also not transcriptionally active in the Orobanchaceae family^27,28^. Secondly, nearly a quarter of the discovered *PaPYL4* alleles are likely to encode incomplete proteins caused by small insertions or deletions. Amongst the twelve different alleles with a full coding sequence and evaluated for interaction with co-receptors in the presence of a range of ABA concentrations, only *PaPYL4* exhibited reduced affinity to ABA co-receptors. The presence of numerous inactive alleles, together with the high proportion of alleles which most probably encode non-functioning proteins, is a strong indication of a relaxed selection of *PaPYL4*. As with *PaPYL4*, the *PaPYL5* coding sequence obtained from the PPGP database, also seemed to be the product of low selective pressure, which enabled vast mutation of a highly conserved region, including the “latch”, one of two surface loops that bind ABA and co-receptors.

Nonetheless, ABA clearly plays a major regulatory role in *P*. *aegyptiaca* seed dormancy, and likely in other parts of the life cycle, as could be deduced from ABA receptor and co-receptor transcription throughout multiple developmental stages. This, together with transcription of ABA biosynthesis components, signifies the prominence of ABA even in an obligate holoparasitic plant. However, many aspects of the role of ABA in *P*. *aegyptiaca* are yet to be understood, especially in the parasitism dynamics with the host plant. The new information gained here could provide a basis for further exploration of ABA involvement in parasitism mechanisms in plants, in general, and of *Orobanche* physiology, in particular. Moreover, structural data of functional *P*. *aegyptiaca* ABA receptors might serve as a scaffold to engineer selective agonists that differentially affect *P*. *aegyptiaca* without harming the host. As ABA inhibits germination and growth, such agonists can provide a new strategy for pest management.

## Methods

### Identification of putative ABA receptor and ABA co-receptor sequences

The PYR1 and ABI1 amino acid sequences were obtained from The *Arabidopsis* Information Resource (Loci AT4G17870.1 and AT4G26080 respectively). These sequences were used to query (TBLASTN) the Parasitic Plant Genome Project database^16^ for homologous nucleic acid sequences in *P*. *aegyptiaca*, *T*. *versicolor* and *S*. *hermonthica*, and the GenBank TSA database for *C*. *pentagona* and *C*. *suaveolens* sequences. Expressed sequence tag (EST) sequences overcoming the e-value 1.0^−10^ threshold, were imported to Geneious^®^ 7.1.9 (https://www.geneious.com) and were assembled using the De Novo Assemble tool (default settings). Open reading frames in the assembled sequences were predicted, translated to amino acids (Standard Code/transl_table 1) and aligned (pairwise MUSCLE alignment, default settings) to PYR1 or ABI1 using Geneious^®^ 7.1.9. Since, in some cases, the genetic material was extracted from tissue connected to the host plant, a basic local alignment search (Standard Nucleotide BLAST) of the PYR1 and ABI1 homologous sequences was conducted using the NCBI database. Cases with high identity with the host species were excluded. In order to identify the tissues and developmental stages in which any ABA receptor or co-receptor were likely to be transcribed, the newly identified sequences were used to query (BLASTN) the PPGP database for homologous nucleic acid sequences in *P*. *aegyptiaca*. In some cases, highly similar sequences were identified via the database nucleotide sequence pairwise alignments, yet some variation (no greater than 5% of the entire sequence) was present. We attributed this to the large allelic variation we observed in our own *in-vitro* experiments, and decided to include these less than perfect matches in the transcription pattern in the presented results.

### Phylogenetic analysis

Phylogenetic trees were inferred using the MEGA6.06 software^29^, based on the MUSCLE method^30^ using the UPGMB clustering method. The START domain of AtPYR1 (38–172) was used as a reference for identification of the START domain of other proteins. The evolutionary history was inferred using the Neighbor-Joining method^31^. Percentage of 1000 replicate trees in which the sequences clustered together in the bootstrap test, is shown next to the branches^32^.

### Sources of P. aegyptiaca RNA and tissue samples

For the initial cloning, *P*. *aegyptiaca* tissues (tubercles, young shoots and flowers) were harvested from plants originating from seeds collected in Ramat-David (Israel) in July 2014. The plants were grown with and parasitized onto tomato cultivar *Solanum Lycopersicum* MP-1 sp. Total RNA was extracted from tubercles, young shoots and flowers using the Spectrum^TM^ Plant Total RNA Kit (Sigma-Aldrich, catalog number STRN50), according to the manufacturer’s instructions. The RNA samples, frozen (−20 °C) tissue and source seeds were kindly provided by Dr. Radi Aly of the Newe Ya’ar Research Center (ARO). cDNA was synthesized from the RNA samples using the SuperScript^®^ III First-Strand Synthesis System for RT-PCR (Invitrogen, catalog number 18080051), according to the manufacturer’s instructions.

### Growth conditions of *P*. *aegyptiaca*

*P*. *aegyptiaca* seeds were mixed in soil (8 g seeds per 1 L soil) and transferred to 4 L pots, into which two-week-old tomato cultivar *Solanum Lycopersicum* M82 sp were planted. The inoculated plants were grown under greenhouse conditions (natural day length, 25 °C/20 °C day/night temperature). The first *P*. *aegyptiaca* flowers broke soil during the third month of the growing period. Tissue samples of the flowers and the stems were collected during the following month, and stored at −80 °C.

### DNA extraction and amplification

*P*. *aegyptiaca* tissue samples were ground to powder using a TissueLyser II (QIAGEN). Samples were mixed with 600 µl DNA extraction buffer and incubated at 65 °C, for 30 min. Chloroform (600 µl) was then added to the samples, which were then centrifuged at 20,000 RCF, for 2 min. The upper phase was isolated and mixed with 600 µl chloroform and centrifuged at 20,000 RCF, for 2 min. The upper phase was isolated again, mixed with isopropanol at a 2:3 ratio, and stored for a least 30 min, at −20 °C. The samples were then centrifuged at 20,000 RCF, for 30 min. The supernatant was discarded and the pellet was washed (not resuspended) with 600 µl cold (−20 °C) 70% ethanol. The samples were then centrifuged at 20,000 RCF, for 5 min, and the supernatant was discarded. In order to remove residual ethanol, the samples were incubated at 60 °C, until the pellet fully dried. The pellet was then resuspended in water.

Selected genes were amplified using Phusion^®^ High-Fidelity DNA Polymerase (New England BioLabs, catalog number M0530L), according to the manufacturer’s instructions. All gene primers were designed using Primer3 version 2.3.4, via Geneious^®^ 7.1.9, and are listed in Table S1.

### Yeast-based receptor activation assays

The coding sequences of *PaPYL4-8* were fused to the GAL4 DNA-binding domain (GBD) coding sequence, by Gibson assembly (New England BioLabs protocol E5510), in a pBD-GAL4 CAM vector (Clontech), restricted by SalI and EcoRI. The coding sequences of *PaABIL1-2*, *PaPP2CAL1-3*, *ABI1 – AT4G26080*, *ABI1*^*G180D*^ *(abi1-1)*, *ABI2 – AT5G57050*, *ABI2*^*G168D*^ *(abi2-1)* and *HAB1 – AT1G72770* were fused to the GAL4-activating domain (GAD) coding sequence by ligation with pACT2 (Clontech), restricted by MfeI and XmaI. The assembled and ligated vectors were cloned and propagated in *Escherichia coli* (DH5α) and then transferred to *Saccharomyces cerevisiae* strain Y190. Transformed yeast were selected for vector presence on synthetic defined medium (SD) lacking leucine and tryptophan. Interaction between PaPYL4-8 and PP2Cs was detected by inculcating individual clones onto plates supplemented with 0.1–10 µM ABA (Biosynth, Switserland) or 0.1% DMSO as solvent control (mock) (48 h, at 30 °C) and then monitoring β-galactosidase reporter gene expression levels through X-Gal staining, as described by Park *et al*. (2009). The assay, for all clones was repeated at least three times.

### *In-vitro* receptor activation assay/phosphatase inhibition assay

#### Protein expression

The *PaPYL4*.*1*, *PaPYL4*.*2* and *PaPYL5* coding sequences were cloned into the pSUMO vector. The *PaPYL7*, *8* and *PaABIL1* coding sequences were Gibson-assembled into a modified pGEX-4T-1 vector (Δ863–893). The constructs were transformed into *E*. *coli* BL21 (DE3) pLysS bacteria by heat-shock. Protein purification was performed with the methods described in Pri-Tal *et al*.^22^.

#### Phosphatase inhibition assay

The inhibitory effect of PaPYL4.1, 4.2, 7 and PaPYL8 on PaABIL1 was measured by reduction in ability of the phosphatase to catalyze the hydrolysis of p-nitrophenyl phosphate (pNPP). The hydrolysis product, p-nitrophenol, is chromogenic and can be detected via spectrophotometry (wavelength of maximum absorbance is 405 nm). Each 100 µl reaction contained 120 nM GST-PaABIL1 with 360 nM 6XHIS-SUMO-PaPYL4.1 or 6XHIS-SUMO-PaPYL5 or 200 nM GST-PaABIL1 with 600 nM GST-PaPYL7 or GST-PaPYL8, in 33 mM Tris·acetate, pH 7.9, 66 mM potassium acetate, 0.1% (w/v) BSA, 10 mM MnCl2, 0.1% (v/v) 2-mercaptoethanol and 50 mM pNPP. Each reaction was supplemented with ABA, dissolved in DMSO (0.1, 0.5, 5 µM), or with 1% (v/v) DMSO only (mock). One of the reactions did not contain any receptor, in order to measure the basal activity of the phosphatase. The hydrolysis reaction was measured every 60 sec, for 20 min, for 6XHIS-SUMO-PaPYL4.1 and 6XHIS-SUMO-PaPYL5, and every 30 sec, for 30 min, for GST-PaPYL7 and GST-PaPYL8. Phosphatase activity was calculated by averaging two technical repetitions. Phosphatase activity levels are presented as a percentage of the phosphatase activity in the presence of a receptor without ABA (mock). This assay was reproduced with two independent protein preparations.

## Supplementary information

Supplementary figures and tables
