# Supplementary material for: ABA signaling components in Phelipanche aegyptiaca
Source: Sci Rep. 2019 Apr 24;9:6476. doi: 10.1038/s41598-019-42976-3 (PMC6482195; doi:10.1038/s41598-019-42976-3)
Supplement: Supplementary file 1 — Supplementary figures and tables [file 41598_2019_42976_MOESM1_ESM.pdf]

ABA signaling components in *Phelipanche aegyptiaca*

Gil Wiseglass[ǂ], Oded Pri-Tal[ǂ], Assaf Mosquna*

The Robert H. Smith Institute of Plant Sciences and Genetics in Agriculture, the Hebrew University of Jerusalem, Rehovot, 7610001, Israel

[ǂ]These authors contributed equally to this work.

[*]Correspondence to assaf.mosquna@mail.huji.ac.il

Supplementary information

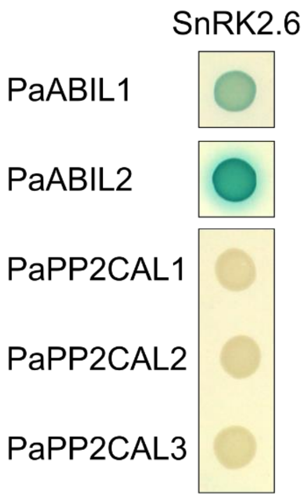

Figure S1. PaABIL1 and PaABIL2 interact with the downstream ABA pathway element SnRK2.6. Activating domain-fused PaABIL1, 2 and PaPP2CAL1, 2, 3 interact with binding domain-fused SnRK2.6 (AT4G33950).

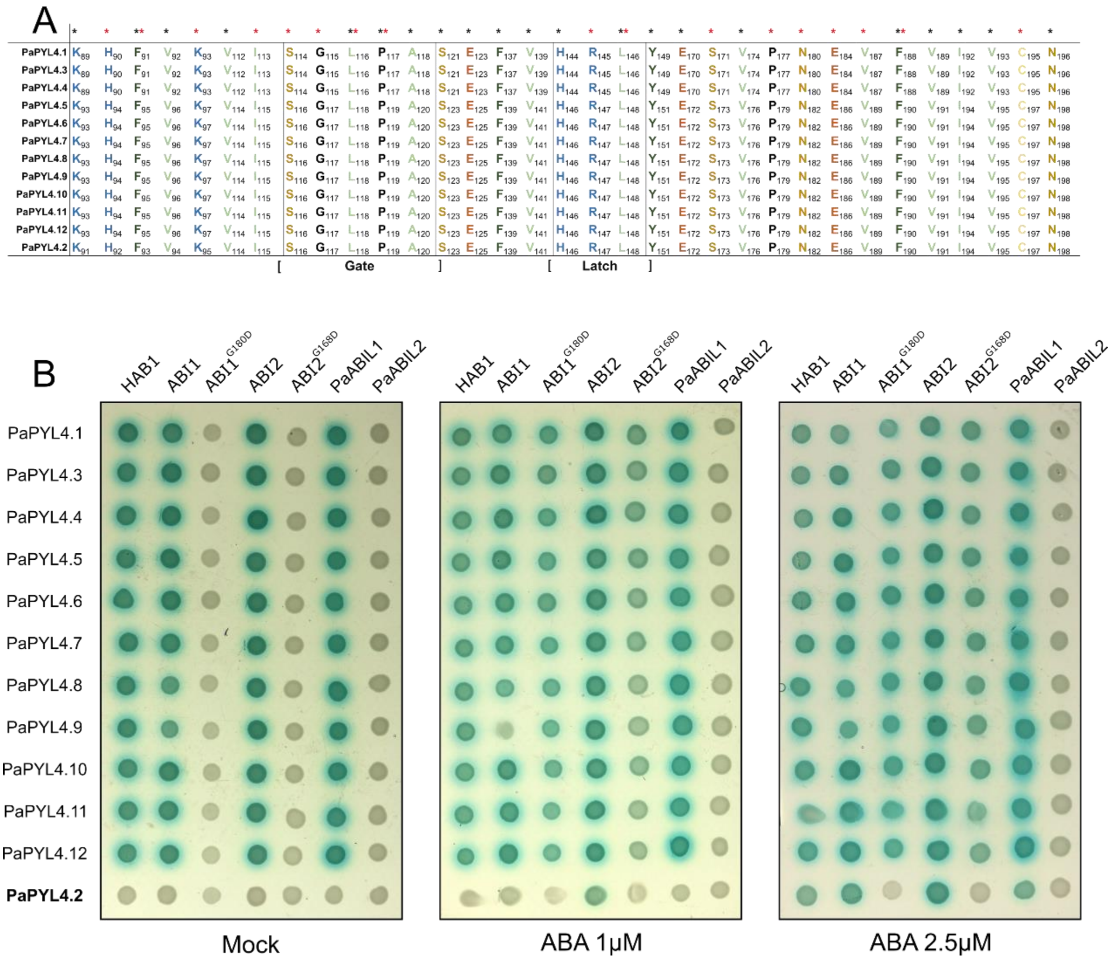

Figure S2. Low ABA sensitivity of PaPYL4.2 does not derive from known ABA/PP2C-interacting residues. (**a**) Alignment of residues which interact with ABA (black asterisks) or HAB1 (red asterisks), as determined by the crystal structures of PYL2-ABA[13] and PYR1-HAB1[17]. Amino acid sequences are color-coded according to side chain characteristics. (**b**) Interaction between binding domain-fused PaPYL4.1-12 and activating domain-fused HAB1, ABI1, ABI1^{G180D}, ABI2, ABI2^{G168D}, PaABIL1 or PaABIL2 in the presence of Mock (0.1% DMSO), or 1 or 2.5 μM ABA.

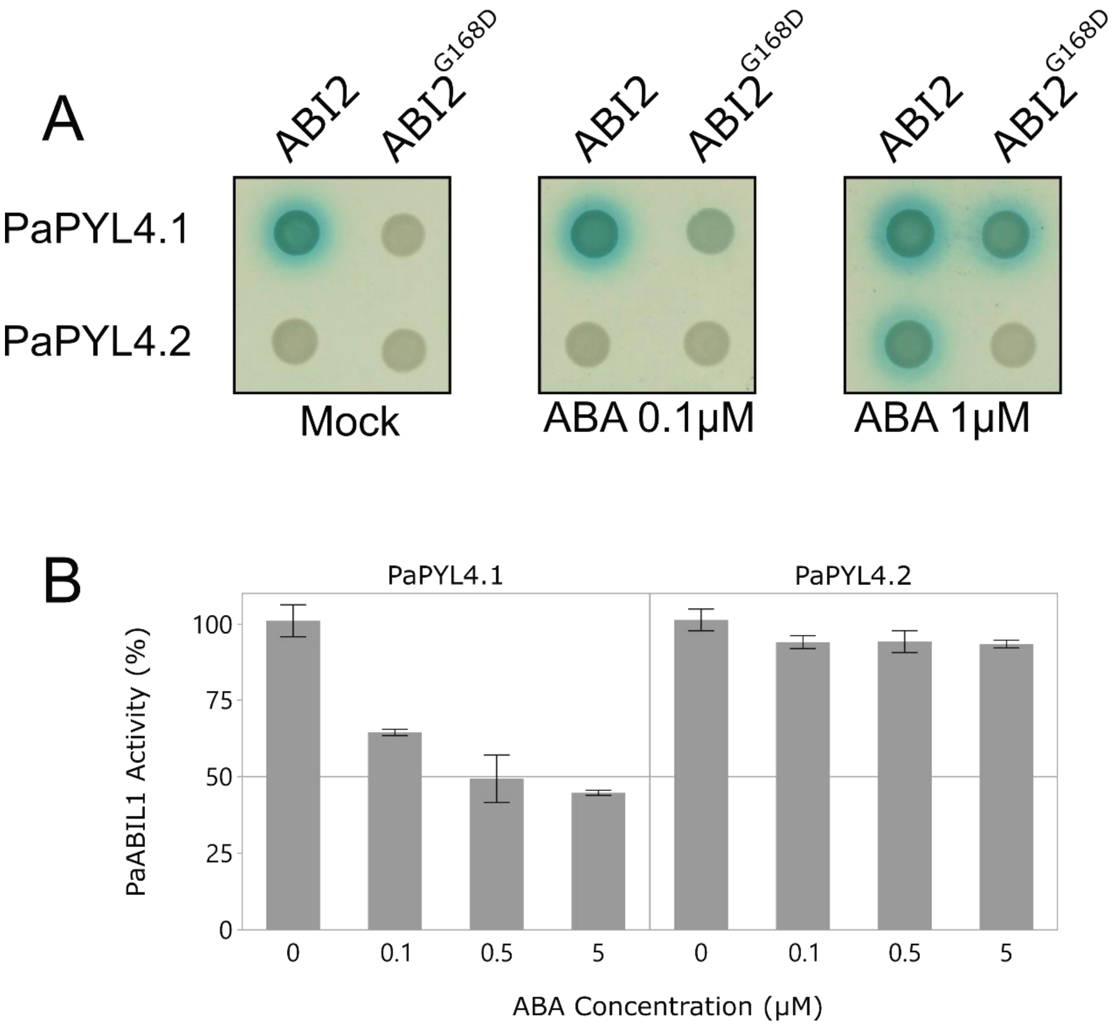

Figure S3. Allelic variation in PaPYL4 affects its interaction with co-receptors. (a) Interaction between binding domain-fused PaPYL4.1 and PaPYL4.2 and activating domain-fused *A. thaliana* ABA co-receptor ABI2 and its mutant $ABI2^{G168D}$, in the presence of Mock (0.1% DMSO), 0.1 µM or 1 µM ABA. **(b)** Recombinant GST-PaABIL1 activity is inhibited by 6XHIS-SUMO-PaPYL4.1 but not by PaPYL4.2. Recombinant GST-PaABIL1 activity (in %) was measured in the presence of recombinant 6XHIS-SUMO-PaPYL4.1 or 6XHIS-SUMO-PaPYL4.2. The reaction contained 120nM GST-PaABIL1 with 360nM receptor and was supplemented with increasing concentrations of ABA (0, 0.1, 0.5 and 5 µM). The activity of two technical replicates of GST-ABIL1 was measured every 60 sec, for 30 min. All calculations were performed in the linear phase of the reaction. The values represent percent activity compared with GST-PaABIL1 without receptors or without ABA (Mock). Data shown for 6XHIS-SUMO-PaPYL4.1 are the same data shown in Figure 5. Bars represent single standard deviation.

Table S1. Genes and oligonucleotide sequences used in this work

Name	Sequence
PaPYL4.1	ATGAAAATGTCTCCCAATCGACCCAAATCCTCCCTTCTACTCCAAAGAATCAACGAAACACCCACCACCACCGCCGCCTCCGCCGCGTGCAAACAACAAGCCTACCGCCACCCCACCGCCGCCCATGTCCCCGA<br>TCACGTCTCCCGGCACCACAACCACCCCGTCGGCCCCAACCAGTGCTGCTCCGCCGTGTCCCAGCACGTCTCCGCCCCCGTCCCCGCGGTCTGGTCCATCGTCCGCCGCTTCGACAACCCCCAGGCGTACAAGC<br>ACTTCGTCAAGACGTGCGACGTCATCCGCGGCCACGGCGACGTCGGCTCGCTCCGCGAGGTCCGCGTCATCTCCGGTCTGCCGGCGGTCAGCAGCACAGAGCGGCTCGAGATCCTCGACGACGAGAGCCACGT<br>CCTCAGCTTCAGCGTCGTCGGCGGGGACCACCGCCTGGCCAACTACCGATCCGTCACCACACTGCACGAGGCACCGACCAACAGCGGAGGCGGGACGGTGGTCGTGGAGTCGTACGTCGTCGACGTGCCGCAG<br>GGTAACACCAAGGAGGAAACGTGCGTGTTCGTCGACACCATTGTCAAATGTAATCTTCATTCCTTAGCTCAGATCTCAGAGAATTTAGCAAGAAGAAACCCTTCTCCATAA
PaPYL4.2	ATGAAAATGTCTCCCAATCGACCCAAATCCTCCCTTCTACTCCAAAGAATCAACGAAACACCCACCACCACCGCCGCCTCCGCTGCCGTCGCGTGCAAACATCAAGCCTACCGCCACCCCACCGCCGCCCATGT<br>CCCCGATCACGTCTCCCGGCACCACAACCACCCCGTCGGCCCCAACCAGTGCTGCTCCGCCGTGTCCCAGCACGTCTCCGCCCCCGTCCCCGCGGTCTGGTCCATCGTCCGCCGATTCGACAACCCACAGGCGT<br>ACAAGCACTTCGTCAAGACGTACGACGTCATCCGAGGCGACGGCGACGTCGGCTCGCTCCGCGAGGTCCGCGTCATCTCCGGTCTGCCGGCGGTCAGCAGCACCGAGCGGCTCGAGATCCTCGACGACGAGCG<br>CCACGTCCTCAGCTTCAGCGTCGTCGGAGGGGACCATCGCCTGGCCAACTACCGATCCGTCACTACACTGCACGAGGCACCGACCAACAGCGGAGGCGGGACGGTGGTCGTGGAGTCGTACGTCGTCGACGTG<br>CCGCAGGGTAACACCAAGGAGGAAACATGCGTGTTCGTCGACACCATTGTCAAATGTAATCTTCATTCCTTAGCTCAGATCTCGGAGAATTTAGCAAGAAGAAACCCTTCTCCATAA
PaPYL4.3	ATGAAAATGTCTCCCAATCGACCCAAATCCTCCCTTCTACTCCAAAGAATCAACGAAACACCCACCACCACCGCCGCCTCCGCCGCGTGCAAACATCAAGCCTACCGCCACCCCACCGCCGCCCATGTCCCCGA<br>TCACGTCTCCCGGCACCACAACCACCCCGTCGGCCCCAACCAGTGCTGCTCCGCCGTGTCCCAGCACGTCTCCGCCCCCGTCCCCGCGGTCTGGTCCATCGTCCGCCGCTTCGACAACCCACAGGCGTACAAGC<br>ACTTCGTCAAGACGTGCGACGTCATCCGAGGCGACGGCGACGTCGGCTCGCTCCGCGAGGTCCGCGTCATCTCCGGTCTGCCGGCGGTCAGCAGCACCGAGCGGCTCGAGATCCTCGACGACGAGCGCCACGT<br>CCTCAGCTTCAGCGTCGTCGGAGGGGACCATCGCCTGGCCAACTACCGATCCGTCACTACACTGCACGAGGCACCGACCAACAGCGGAGGCGGGACGGTGGTCGTGGAGTCGTACGTCGTCGACGTGCCGCAG<br>GGTAACACCAAGGAGGAAACATGCGTGTTCGTCGACACCATTGTCAAATGTAATCTTCATTCCTTAGCTCAGATCTCAGAGAATTTAGCAAGAAGAAACCCTTCTCCATAA
PaPYL4.4	ATGAAAATGTCTCCCAATCGACCCAAATCCTCCCTTCTACTCCAAAGAATCAACGAAACACCCACCACCACCGCCGCCTCCGCCGCGTGCAAACATCAAGCCTACCGCCACCCCACCGCCGCCCATGTCCCCGA<br>TCACGTCTCCCGGCACCACAACCACCCCGTCGGCCCCAACCAGTGCTGCTCCGCCGTGTCCCAGCACGTCTCCGCCCCCGTCCCCGCGGTCTGGTCCATCGTCCGCCGCTTCGACAACCCACAGGCGTACAAGC<br>ACTTCGTCAAGACGTGCGACGTCATCCGAGGCGACGGCGACGTCGGCTCGCTCCGCGAGGTCCGCGTCATCTCCGGTCTGCCGGCGGTCAGCAGCACCGAGCGGCTCGAGATCCTCGACGACGAGCGCCACGT<br>CCTCAGCTTCAGCGTCGTCGGAGGGGACCATCGCCTGGCCAACTACCGATCCGTCACTACACTGCACGAGGCACCGACCAACAGCGGAGGCGGGACGGTGGTCGTGGAGTCGTACGTCGTCGACGTGCCGCAG<br>GGTAACACCAAGGAGGAAACATGCGTGTTCGTCGACACCATTGTCAAATGTAATCTTCATTCCTTAGCTCAGATCTCAGAGAATTTAGCAAGAAGAAACCCTTCTCCATAA
PaPYL4.5	ATGAAAATGTCTCCCAATCGACCCAAATCCTCCCTTCTACTCCAAAGAATCAACGAAACACCCACCACCACCGCCGCCTCCGCTGCCGTCGCGTGCAAACATCAAGCCTACCGCCACCCCACCGCCGCCCATGT<br>CCCCGATCACGTCTCCCGGCACCACAACCACCCCGTCGGCCCCAACCAGTGCTGCTCCGCCGTGTCCCAGCACGTCTCCGCCCCCGTCCCCGCGGTCTGGTCCATCGTCCGCCGATTCGACAACCCACAGGCGT<br>ACAAGCACTTCGTCAAGACGTGCGACGTCATCCGAGGCGACGGCGACGTCGGCTCGCTCCGCGAGGTCCGCGTCATCTCCGGTCTGCCGGCGGTCAGCAGCACCGAGCGGCTCGAGATCCTCGACGACGAGCG<br>CCACGTCCTCAGCTTCAGCGTCGTCGGAGGGGACCATCGCCTGGCCAACTACCGATCCGTCACTACACTGCACGAGGCACCGACCAACAGCGGAGGCGGGACGGTGGTCGTGGAGTCGTACGTCGTCGACGTG<br>CCGCAGGGTAACACCAAGGAGGAAACATGCGTGTTCGTCGACACCATTGTCAAATGTAATCTTCATTCCTTAGCTCAGATCTCGGAGAATTTAGCAAGAAGAAACCCTTCTCCATAA
PaPYL4.6	ATGAAAATGTCTCCNAATCGACCCAAATCCTCCCTTCTACTCCAAAGAATCAACGAAACACCCACCACCACCGCCGCCTCCGCTGCCGTCGCGTGCAAATATCAAGCCTACCGCCACCCCACCGCCGCCCATGT<br>CCCCGATCACGTCTCCCGGCACCACAACCACCCCGTCGGCCCCAACCAGTGCTGCTCCGCCGTGTCCCAGCACGTCTCCGCCCCCGTCCCCGCGGTCTGGTCCATCGTCCGCCGATTCGACAACCCACAGGCGT<br>ACAAGCACTTCGTCAAGACGTGCGACGTCATCCGAGGCGACGGCGACGTCGGCTCGCTCCGGGAGGTCCGCGTCATCTCCGGTCTGCCGGCGGTTAGCAGCACCGAGCGGCTCGAGATCCTCGACGACGAGCG<br>CCACGTCCTCAGCTTCAGCGTCGTCGGCGGGGATCACCGCCTGGCCAACTACCGATCCGTCACCACACTGCACGAGGCGTCGACNAACGGCGGAGGCGGAACGGTGGTCGTGGAGTCGTACGTCGTCGACGTG<br>CCGCAGGGTAACACCAAGGAGGAAACATGCGTGTTCGTCGACACCATTGTCAAATGTAATCTTCATTCCTTAGCTCAGATCTCAGAGAATTTAGCAAGAAGAAACCCTTCTCCATAA
PaPYL4.7	ATGAAAATGTCTCCNAATCGACCCAAATCCTCCCTTCTACTCCAAAGAATCAACGAAACACCCACCACCACCGCCGCCTCCGCTGTCGTCGCGTGCAAATATCAAGCCTACCGCCACCCCACCGCCGCCCATGT<br>CCCCGATCACGTCTCCCGGCACCACAACCACCCCGTCGGCCCCAACCAGTGCTGCTCCGCCGTGTCCCAGCACGTCTCCGCCCCCGTCCCCGCGGTCTGGTCCATCGTCCGCCGCTTCGACAACCCCCAGGCGTA<br>CAAACACTTCGTCAAGACGTGCGACGTCATCCGCGGCCACGGCGACGTCGGCTCGCTCCGGGAGGTCCGCGTCATCTCCGGTCTGCCGGCGGTCAGCAGCACCGAGCGGCTCGAGATCCTCGACGACGAGCGC<br>CACGTCCTCAGCTTCAGCGTCGTCGGAGGGGACCATCGCCTGGCCAACTACCGATCCGTCACCACACTGCACGAGGCACCGACCAACAGCGGAGGCGGGACGGTGGTCGTGGAGTCGTACGTCGTCGACGTGC<br>CGCAGGGTAACACGAAGGAGGAAACGTGCGTGTTCGTCGACACCATTGTCAAATGTAACCTTCATTCCTTAGCTCAGATCTCGGAGAATTTAGCAAGAAGAAACCCTTCTCCATAA
PaPYL4.8	ATGAAAATGTCTCCCAATCGACCCAAATCCTCCCTTCTACTCCAAAGAATCAACGAAACACCCACCACCACCGCCGCCTCCGCTGCCGTCGCGTGCAAACATCAAGCCTACCGCCACTCCACCGCCGCCCATGT<br>CCCCGATCACGTCTCCCGGCACCACAACCACCCCCTCGGCCCCAACCAGTGCTGCTCCGCCGTGTCCCAGCACGTCTCCGCCCCCGTCCCCGCGGTCTGGTCCATCGTCCGCCGATTCGACAACCCACAGGCGTA<br>CAAACACTTCGTCAAGACGTGCGACGTCATCCGAGGCGACGGCGACGTCGGCTCGCTCCGCGAGGTCCGCGTCATCTCCGGTCTGCCGGCGGTCAGCAGCACCGAGCGGCTCGAGATCCTCGACGACGAGCGC<br>CACGTCCTCAGCTTCAGCGTCGTCGGCGGGGATCACCGCCTGGCCAACTACCGATCCGTCACCACACTGCACGAGGCACCGACCAACAGCGGAGGCGGGACGGTGGTCGTGGAGTCGTACGTCGTCGACGTGC<br>CGCAGGGTAACACCAAGGAGGAAACATGCGTGTTCGTCGACACCATTGTCAAATGTAATCTTCATTCCTTAGCTCAGATCTCGGAGAATTTAGCAAGAAGAAACCCTTCTCCATAA
PaPYL4.9	ATGAAAATGTCTCCCAATCGACCCAAATCCTCCCTTCTACTTCAAAGAATCAACGAAACACCCCCCACCACCGCCGCCTCCGCTGCCGTCGCGTGCAAACATCAAGCCTACCGCCACCCCATCGCCGCCCATGT<br>CCCCGATCACGTCTCCCGGCACCACAACCACCCCCTCGGCCCCAACCAGTGCTGCTCCGCCGTGTCCCAGCACGTCTCCGCCCCCGTCCCCGCGGTCTGGTCCATCGTCCGCCGATTCGACAACCCACAGGCGTA<br>CAAACACTTCGTCAAGACGTGCGACGTCATCCGAGGCGACGGCGACGTCGGCTCGCTCCGCGAGGTCCGCGTCATCTCCGGTCTGCCGGCGGTCAGCAGCACCGAGCGGCTCGAGATCCTCGACGACGAGCGC<br>CACGTCCTCAGCTTCAGCGTCGTCGGCGGGGATCACCGCCTGGTCAACTACCGATCCGTCACCACACTGCACGAGGCACCGACCAACAGCGGAGGCGGGACGGTGGTCGTGGAGTCGTACGTCGTCGACGTGC<br>CGCAGGGTAACACCAAGGAGGAAACATGCGTGTTCGTCGACACCATTGTCAAATGTAATCTTCATTCCTTAGCTCAGATCTCGGAGAATTTAGCAAGAAGAAACCCTTCTCCATAA
PaPYL4.10	ATGAAAATGTCTCCNAATCGACCCAAATCCTCCCTTCTACTTCAAAGAATCAACGAAACACCCACCACCACCGCCGCCTCCGCTGCCGTCGCGTGCAAACATCAAGCCTACCGCCACCCCACCGCCGCCCATGT<br>CCCCGATCACGTCTCCCGGCACCACAACCACCCCGTCGGCCCCAACCAGTGCTGCTCCGCCGTGTCCCAGCACGTCTCCGCCCCCGTCCCCTCGGTCTGGTCCATCGTCCGCCGCTTCGACAACCCACAGGCGTA<br>CAAACACTTCGTCAAGACGTGCGACGTCATCCGAGGCGACGGCGACGTCGGCTCGCTCCGCGAGGTCCGCGTCATCTCCGGTCTGCCGGCGGTCAGCAGCACCGAGCGGCTCGAGATCCTCGACGACGAGCGC<br>CACGTCCTCAGCTTCAGCGTCGTCGGCGGGGATCACCGCCTGGCCAACTACCGATCCGTCACCACACTGCACGAGGCACCGACCAACAGCGGAGGCGGGACGGTGGTCGTGGAGTCGTACGTCGTCGACGTGC<br>CGCAGGGTAACACCAAGGAGGAAACATGCGTGTTCGTCGACACCATTGTCAAATGTAATCTTCATTCCTTAGCTCAGATCTCGGAGAATTTAGCAAGAAGAAACCCTTCTCCATAA
PaPYL4.11	ATGAAAATGTCTCCCAATCGACCCAAATCCTCCCTTCTACTTNAAAGAATCAACGAAACACCCACCACCACCGCCGCCTCCGCTGCCGTCGCGTGCAAACATCAAGCCTACCGCCACCCCACCGCCGCCCATGT<br>CCCCGATCACGTCTCCCGGCACCACAACCACCCCCTCGGCCCCAACCAGTGCTGCTCCGCCGTGTCCCAGCACGTCTCCGCCCCCGTCCCCGCGGTCTGGTCCATCGTCCGCCGATTCGACAACCCACAGGCGTA<br>CAAACACTTCGTCAAGACGTGCGACGTCATCCGAGGCGACGGCGACGTCGGCTCGCTCCGCGAGGTCCGCGTCATCTCCGGTCTGCCGGCGGTCAGCAGCACCGAGCGGCTCGAGATCCTCGACGACGAGCGC<br>CACGTCCTCAGCTTCAGCGTCGTCGGAGGGGACCACCGCCTGACCAACTACCGATCCGTCACCACACTGCACGAGGCACCGACCAACGGCGGAGGCGGAACGGTGGTCGTGGAGTCGTACGTCGTCGACGTGC<br>CGCAGGGTAACACGAAGGAGGAAACGTGCGTGTTCGTCGACACCATTGTCAAATGTAATCTTCATTCCTTAGCTCAGATCTCGGAGAATTTAGCAAGAAGAAACCCTTCTCCATAA
PaPYL4.12	ATGAAAATGTCTCCCAATCGACCCAAATCCTCCCTTCTACTCCAAAGAATCAACGAAACACCCACCACCACCGCCGCCTCCGCTGCCGTCGCGTGCAAACATCAAGCCTACCGCCACCCCACCGCCGCCCATGT<br>CCCCGATCACGTCTCCCGGCACCACAACCACCCCGTCGGCCCCAACCAGTGCTGCTCCGCCGTGTCCCAGCACGTCTCCGCCCCCGTCCCCTCGGTCTGGTCCATCGTCCGCCGCTTCGACAACCCACAGGCGTA<br>CAAGCACTTCGTCAAGACGTGCGACGTCATCCGAGGCGACGGCGACGTCGGCTCGCTCCGCGAGGTCCGCGTCATCTCCGGTCTGCCGGCGGTCAGCAGCACCGAGCGGCTCGAGATCCTCGACGACGAGCGC<br>CACGTCCTCAGCTTCAGCGTCGTCGGAGGGGACCACCGCCTGGCCAACTACCGATCCGTCACCACACTGCACGAGGCACCGACCAACGGCGGAGGCGGAACGGTGGTCGTGGAGTCGTACGTCGTCGACGTGC<br>CGCAGGGTAACACGAAGGAGGAAACGTGCGTGTTCGTCGACACCATTGTCAAATGTAATCTTCATTCCTTAGCTCAGATCTCGGAGAATTTAGCAAGAAGAAACCCTTCTCCATAA
PaPYL4.13	ATGAAAATGTCTCCNAATCGACCCAAATCCTCCCTTCTACTCCAAAGAATCAACGAAACACCCACCACCACCGCCGCCTCCGCTGTCGTCGCGTGCAAATATCAAGCCTACCGCCACCCCACCGCCGCCCATGT<br>CCCCGATCACGTCTCCCGGCACCACAACCACCCCGTCGGCCCCAACCAGTGCTGCTCCGCCGTGTCCCAGCACGTCTCCGCCCCCGTCCCCGCGGTCTGGTCCATCGTCCGCCGCTTCGACAACCCCCAGGCGTA<br>CAAACACTTCGTCAAGACGTGCGACGTCATCCGCGGCCACGGCGACGTCGGCTCGCTCCGGGAGGTCCGCGTCATCTCCGGTCTGCCGGCGGTCAGCAGCACCGAGCGGCTCGAGATCCTCGACGACGAGCGC<br>CACGTCCTCAGCTTCAGCGTCGTCGGAGGGGACCATCGCCTGGCCAACTACCGATCCGTCACCACACTGCACGAGGCACCGACCAACAGCGGAGGCGGGACGGTGGTCGTGGAGTCGTACGTCGTCGACGTGC<br>CGCAGGGTAACACGAAGGAGGAAACGTGCGTGTTCGTCGACACCATTGTCAAATGTAACCTTCATTCCTTAGCTCAGATCTCGGAGAATTTAGCAAGAAGAAACCCTTCTCCATAA
PaPYL4.14	ATGAAAATGTCTCCCAATCGACCCAAATCCTCCCTTCTACTCCAAAGAATCAACGAAACACCCACCACCACCGCCGCCTCCGCTGCCGTCGCGTGCAAATATCAAGCCTACCGCCACCCCACCGCCGCCCATGT<br>CCCCGATCACGTCTCCCGGCACCACAACCACCCCGTCGGCCCCAACCAGTGCTGCTCCGCCGTGTCCCAGCACGTCTCCGCCCCCGTCCCCGCGGTCTGGTCCATCGTCCGCCGATTCGACAACCCACAGGCGT<br>ACAAGCACTTCGTCAAGACGTGCGACGTCATCCGAGGCGACGGCGACGTCGGCTCGCTCCGGGAGGTCCGCGTCATCTCCGGTCTGCCGGCGGTTAGCAGCACCGAGCGGCTCGAGATCCTCGACGACGAGCG<br>CCACGTCCTCAGCTTCAGCGTCGTCGGCGGGGATCACCGCCTGGCCAACTACCGATCCGTCACCACACTGCACGAGGCGTCGACCAACGGCGGAGGCGGAACGGTGGTCGTGGAGTCGTACGTCGTCGACGTG<br>CCGCAGGGTAACACCAAGGAGGAAACATGCGTGTTCGTCGACACCATTGTCAAATGTAATCTTCATTCCTTAGCTCAGATCTCGGAGAATTTAGCAAGAAGAAACCCTTCTCCATAA
PaPYL4.15	ATGAAAATGTCTCCCAATCGACCCAAATCCTCCCTTCTACTTCAAAGAATCAACGAAACACCCACCACCACCGCCGCCTCCGCTGCCGTCGCGTGCAAACATCAAGCCTACCGCCACCCCACCGCCGCCCATGT<br>CCCCGATCACGTCTCCCGGCACCACAACCACCCCGTCGGCCCCAACCAGTGCTGCTCCGCCGTGTCCCAGCACGTCTCCGCCCCCGTCCCCTCGGTCTGGTCCATCGTCCGCCGCTTCGACAACCCACAGGCGTA<br>CAAACACTTCGTCAAGACGTGCGACGTCATCCGAGGCGACGGCGACGTCGGCTCGCTCCGCGAGGTCCGCGTCATCTCCGGTCTGCCGGCGGTCAGCAGCACCGAGCGGCTCGAGATCCTCGACGACGAGCGC<br>CACGTCCTCAGCTTCAGCGTCGTCGGCGGGGATCACCGCCTGGCCAACTACCGATCCGTCACCACACTGCACGAGGCACCGACCAACAGCGGAGGCGGGACGGTGGTCGTGGAGTCGTACGTCGTCGACGTGC<br>CGCAGGGTAACACCAAGGAGGAAACATGCGTGTTCGTCGACACCATTGTCAAATGTAATCTTCATTCCTTAGCTCAGATCTCGGAGAATTTAGCAAGAAGAAACCCTTCTCCATAA
PaPYL4.16	ATGAAAATGTCTCCCAATCGACCCAAATCCTCCCTTCTACTTCAAAGAATCAACGAAACACCCACCACCACCGCCGCCTCCGCTGCCGTCGCGTGCAAACATCAAGCCTACCGCCACCCCACCGCCGCCCATGT<br>CCCCGATCACGTCTCCCGGCACCACAACCACCCCCTCGGCCCCAACCAGTGCTGCTCCGCCGTGTCCCAGCACGTCTCCGCCCCCGTCCCCGCGGTCTGGTCCATCGTCCGCCGATTCGACAACCCACAGGCGTA<br>CAAACACTTCGTCAAGACGTGCGACGTCATCCGAGGCGACGGCGACGTCGGCTCGCTCCGCGAGGTCCGCGTCATCTCCGGTCTGCCGGCGGTCAGCAGCACCGAGCGGCTCGAGATCCTCGACGACGAGCGC

	CACGTCCTCAGCTTCAGCGTCGTCGGAGGGGACCACCGCCTGACCAACTACCGATCCGTCACCACACTGCACGAGGCACCGACCAACGGCGGAGGCGGAACGGTGGTCGTGGAGTCGTACGTCGTCGACGTGC<br>CGCAGGGTAACACGAAGGAGGAAACGTGCGTGTTCGTCGACACCATTGTCAAATGTAATCTTCATTCCTTAGCTCAGATCTCGGAGAATTTAGCAAGAAGAAACCCTTCTCCATAA
PaPYL4.17	ATGAAAATGTCTCCCAATCGACCCAAATCCTCCCTTCTACTCCAAAGAATCAACGAAACACCCACCACCACCGCCGCCTCCGCTGCCGTCGCGTGCAAACATCAAGCCTACTGCCACCCCACCGCCGCCCATGT<br>CCCCGATCACATCTCCCGGCACCACAACCACCCCCTCGGCCCCAACCAGTGCTGCTCCGCCGTGTCCCAGCACGTCTCCGCCCCCGTCCCCGCGGTCTGGTCCATCGTCCGCCGCTTCGACAACCCACAGGCGTA<br>CAAGCACTTCGTCAAGACGTGCGACGTCATCCGAGGCGACGGCGACGTCGGCTCGCTCCGCGAGGTCCGCGTCATCTCCGGCCTGCCGGCGGTCAGCAGCACCGAGCGGCTCGAGATCCTCGACGACGAGCGC<br>CACGTCCTCAGCTTCAGCGTCGTCGGAGGGGACCACCGCCTGGCCAACTACCGATCCGTCACCACACTGCACGAGGCACCGACCAACAGCGGAGGCGGGACGGTGGTCGTGGAGTCGTACGTCGTCGACGTGC<br>CGCAGGGTAACACGAAGGAGGAAACGTGCGTGTTCGTCGACACCATTGTCAAATGTAATCTTCATTCCTTAGCTCATCTCAGAGAATTTAGCAAGAAGAAACCCTTCTCCATAAAT
PaPYL4.18	ATGTCTCCCAATCGACCCAAATCCTCCCTTCTACTNCAAAGAATCAACGAAACACCCACCACCACCGCCGCCTCCGCTGCCGTCGCGTGCAAATATCAAGCCTACCGCCACCCCACCGCCGCCCATGTCCCCGA<br>TCACGTCTCCCGGCACCACAACCACCCCGTCGGCCCCAACCAGTGCTGCTCCGCCGTGTCCCAGCACGTCTCAGCCCCCGTCCCCGCGGTCTGGTCCATCGTCCGCCGNTTCGACAACCCACAGGCGTACAANC<br>ACTTCGTCAAGACNTGCGACGTCATCCGCGGCGACGGCGACGTCGGCTCGCTCCGCGAGGTCCGCGTCATCTCCGGTCTGCCGGCGGTCAGCAGCACCGAGCGGCTCGAGATCCTCGACGACGAGCGCCACGT<br>CCTCAGCTTCAGCGTCGTCGGNGGGGACCACCGCCTGGCCAACTACCGATCCGTCACCACACTGCACGAGGCACCGACCAACAGCGGAGGCGGGACGGTGGTCGTGGAGTCGTACGTCGTCGACGTGCCGCAG<br>GGTAACACGAAGGAGGAAACGTGCGTGTTCGTCGACACCATTGTCAAATGTAATCTTCATTCCTTAGCTCAGATCTCAGAGAATTTAGCAAGAAGAAACCCTTCTCCATAA
PaPYL4.19	ATGTCTCCCAATCGACCCAAATCCTCCCTTCTACTTCCAAAGAATCAACGAAACACCCACCACCACCGCCGCCTCCGCTGCCGTCGCGTGCAAANATCAAGCCTACCGCCACCCCACCGCCGCCCATGTCCCCG<br>ATCACGTCTCCCGGCACCACAACCACCCCGTCGGCCCCAACCAGTGCTGCTCCGCCGTGTCCCAGCACGTCTCNGCCCCCGTCCCCGCGGTCTGGTCCATCGTCCGCCGNTTCGACAACCCACAGGCGTACAAG<br>CACTTCGTCAAGACGTGCGACGTCATCCGNGGCGACGGCGACGTCGGCTCGCTCCGCGAGGTCCGCGTCATCTCCGGTCTGCCGGCGGTCAGCAGCACCGAGCGGCTCGAGATCCTCGACGACGAGCGCCACG<br>TCCTCAGCTTCAGCGTCGTCGGNGGGGACCANCGCCTGGCCAACTACCGATCCGTCACGACACTGCACGAGGCACCGACCAACAGCGGAGGCGGGACGGTGGTCGTGGAGTCGTACGTCGTCGACGTGCCGCA<br>GGGTAA
PaPYL4.20	ATGTCTCCCAATCGACCCAAATCCTCCCTTCTACTCCAAAGAATCAACGAAACACCCACCACCACCGCCGCCTCCGCTGCCGTCGCGTGCAAANATCAAGCCTACCGCCACCCCACCGCCGCCCATGTCCCCGA<br>TCACGTCNCCCGGCACCACAACCACCCCGTCGGCCCCAACCAGTGCTGCTCCGCCGTGTCCCAGCACGTCTCCGCCCCCGTCCCCGCGGTCTGGTCCATCGTCCGCCGATTCGACAACCCACAGGCGTACAANC<br>ACTTCGTCAAGACGTGCGACGTCATCCGNGGCGACGGCGACGTCGGCTCGCTCCGCGAGGTCCGCGTCATCTCCGGTCTGCCGGCGGTCAGCAGCACCGAGCGGCTCGAGATCCTCGACGACGAGCGCCACGT<br>CCTCAGCTTCAGCGTCGTCGGAGGGGACCANCGCCTGGCCAACTACCGATCCGTCACNACACTGCACGAGGCACCGACCAACAGCGGAGGCGGGACGGTGGTCGTGGAGTCGTACGTCGTCGACGTGCCGCA<br>GGGTAACACNAAGGAGGAAACNTGCGTGTTCGTCGACACCATTGTCAAATGTAATCTTCATTCCTTAGCTCAGATCTCNGAGAATTTAGCAAGAAGAAACCCTTCTCCATAA
PaPYL4.21	ATGTCTCCCAATCGACCCAAATCCTCCCTTCTACTCCAAAGAATCAACGAAACACCCACCACCACCGCCGCCTCCGCTGCCGTCGCGTGCAAANATCAAGCCTACCGCCACCCCACCGCCGCCCATGTCCCCGA<br>TCACGTCTCCCGGCACCACAACCACCCCGTCGGCCCCAACCAGTGCTGCTCCGCCGTGTCCCAGCACGTCTCCGCCCCCGTCCCCGCGGTCTGGTCCATCGTCCGCCGATTCGACAACCCACAGGCGTACAAGC<br>ACTTCGTCAAGACGTGCGACGTCATCCGAGGCGACGGCGACGTCGGCTCGCTCCGCGAGGTCCGCGTCATCTCCGGTCTGCCGGCGGTCAGCAGCACCGAGCGGCTCGAGATCCTCGACGACGAGCGCCACGT<br>CCTCAGCTTCAGCGTCGTCGGAGGGGACCATCGCCTGGCCAACTACCGATCCGTCACTACACTGCACGAGGCACCGACCAACAGCGGAGGCGGGACGGTGGTCGTGGAGTCGTACGTCGTCGACGTGCCGCAG<br>GGTAACACCAAGGAGGAAACATGCGTGTTCGTCGACACCATTGTCAAATGTAATCTTCATTCCTTAGCTCAGATCTCGGAGAATTTAGCAAGAAGAAACCCTTCTCCATAA
PaPYL4.22	ATGTCTCCCAATCGACCCAAATCCTCCCTTCTACTCCAAAGAATCAACGAAACACCCACCACCACCGCCGCCTCCGCTGCCGTCGCGTGCAAANATCAAGCCTACCGCCACCCCACCGCCGCCCATGTCCCCGA<br>TCACGTCTCCCGGCACCACAACCACCCCGTCGGCCCCAACCAGTGCTGCTCCGCCGTGTCCCAGCACGTCTCCGCCCCCGTCCCCGCGGTCTGGTCCATCGTCCGCCGNTTCGACAACCCACAGGCGTACAANC<br>ACTTCGTCAAGACGTGCGACGTCATCCGNGGCGACGGCGACGTCGGCTCGCTCCGCGAGGTCCGCGTCATCTCCGGTCTGCCGGCGGTCAGCAGCACCGAGCGGCTCGAGATCCTCGACGACGAGCGCCACGT<br>CCTCAGCTTCAGCGTCGTCGGAGGGGACCANCGCCTGGCCAACTACCGATCCGTCACNACACTGCACGAGGCACCGACCAACAGCGGAGGCGGGACGGTGGTCGTGGAGTCGTACGTCGTCGACGTGCCGCA<br>GGGTAACACNAAGGAGGAAACNTGCGTGTTCGTCGACACCATTGTCAAATGTAATCTTCATTCCTTAGCTCAGATCTCNGAGAATTTAGCAAGAAGAAACCCTTCTCCATAA
PaPYL4.23	ATGTCTCCCAATCGACCCAAATCCTCCCTTCTACTTCAAAGAATCAACGAAACACCCACCACCACNGCCGCCTCCGCTGCCGTCGCGTGCAAATATCAAGCCTACCGCCACCCCACCGCCGCCCATGTCCCCGA<br>TCACGTCTCCCGGCACCACAACCACCCCGTCGGCCCCAACCAGTGCTGCTCCGCCGTGTCCCAGCACGTCTCCGCCCCCGTCCCCGCGGTCTGGTCCATCGTCCGCCGATTCGACAACCCACAGGCGTACAAGC<br>ACTTCGTCAAGACGTGCGACGTCATCCGAGGCGACGGCGACGTCGGCTCGCTCCGCGAGGTCCGCGTCATCTCCGGTCTGCCGGCGGTCAGCAGCACCGAGCGGCTCGAGATCCTCGACGACGAGCGCCACGT<br>CCTCAGCTTCAGCGTCGTCGGCGGGGACCACCGCCTGGCCAACTACCGATCCGTCACCACACTGCACGAGGCACCGACCAACAGCGGAGGCGGGACGGTGGTCGTGGAGTCGTACGTCGTCGACGTGCCGCAG<br>GGTAACACGAAGGAGGAAACGTGCGTGTTCGTTGACACCATTGTCAAATGTAATCTTCATTCCTTAGCTCAGATCTCGGAGAATTTAGCAAGAAGAAACCCTTCTCCATAA
PaPYL4.24	ATGAAAATGTCTCCCAATCGACCCAAATCCTCCCTTCTACTCCAAAGAATCAACGAAACACCCACCACCACCGCCGCCTCCGCTGCCGTCGCGTGCAAATATCAAGCCTACCGCCACCCCACCGCCGCCCATGT<br>CCCCGATCACGTCACCCGGCACCACAACCACCCCGTCGGCCCCAACCAGTGCTGCTCCGCCGTGTCCCAGCACGTCTCCGCCCCCGTCCCCGCGGTCTGGTCCATCGTCCGCCGCTTCGACAACCCACAGGCGT<br>ACAAACACTTCGTCAAGACGTGCGACGTCATCCGCGGCGACGGCGACGTCGGCTCGCTCCGCGAGGTCCGCGTCATCTCCGGTCTGCCGGCGGTCAGCAGCACCGAGCGGCTCGAGATCCTCGACGACGAGCG<br>CCACGTCCTCAGCTTCAGCGTCGTCGGAGGGGACCACCGCCTGGCCAACTACCGATCCGTCACCACACTGCACGAGGCACCGACCAACAGCGGAGGCGGGACGGTGGTCGTGGAGTCGTACGTCGTCGACGTG<br>CCGCAGGGTAACACGAAGGAGGAAACGTGCGTGTTCGTCGACACCATTGTCAAATGTAATCTTCATTCCTTAGCTCAGATCTCAGAGAATTTAGCAAGAAGAAACCCTTCTCCATAA
PaPYL4.25	ATGTCTCCCAATCGACCCAAATCCTCCCTTCTACTCCAAAGAATCAACGAAACACCCACCACCACCGCCGCCTCCGCTGCCGTCGCGTGCAAANATCAAGCCTACCGCCACCCCACCGCCGCCCATGTCCCCGA<br>TCACGTCTCCCGGCACCACAACCACCCCGTCGGCCCCAACCAGTGCTGCTCCGCCGTGTCCCAGCACGTCTCCGCCCCCGTCCCCNCGGTCTGGTCCATCGTCCGCCGATTCGACAACCCACAGGCGTACAAGC<br>ACTTCGTCAAGACGTGCGACGTCATCCGAGGCGACGGCGACGTCGGCTCGCTCCGNGAGGTCCGCGTCATCTCCGGTCTGCCGGCGGTCAGCAGCACCGAGCGGCTCGAGATCCTCGACGACGAGCGCCACGT<br>CCTCAGCTTCAGCGTCGTCGGNGGGGANCACCGCCTGGCCAACTACCGATCCGTCACCACACTGCACGAGGCNNCGACCAACNGCGGAGGCGGAACGGTGGTCGTGGAGTCGTACGTCGTCGACGTGCCGCA<br>GGGTAACACNAAGGAGGAAACNTGCGTGTTCGTNGACACCATTGTCAAATGTAANCTTCATTCCTTAGCTCAGATCTCNGAGAATTTAGCAAGAAGAAACCCTTCTCCATAA
PaPYL4.26	ATGTCTCCCAATCGACCACAAATCCTCCCTTCTACTTCCAAAGAATCAACGAAACACCCACCACCACCGCCGCCTCCGCTGCCGTCGCGTGCAAACATCAAGCCTACCGCCACCCCACCGCCGCCCATGTCCCC<br>GATCACGTCTCCCGGCACCACAACCACCCCGTCGGCCCCAACCAGTGCTGCTCCGCCGTGTCCCAGCACGTCTCCGCCCCCGTCCCCGCGGTCTGGTCCATCGTCCGCCGNTTCGACAACCCACAGGCGTACAA<br>GCACTTCGTCAAGACGTGCGACGTCATCCGAGGCGACGGCGACGTCGGCTCGCTCCGNGAGGTCCGCGTCATCTCCGGTCTGCCGGCGGTCAGCAGCACCGAGCGGCTCGAGATCCTCGACGACGAGCGCCAC<br>GTCCTCAGCTTCAGCGTCGTCGGAGGGGACCANCGCCTGGCCAACTACCGATCCGTCACTACACTGCACGAGGCACCGACCAACAGCGGAGGCGGGACGGTGGTCGTGGAGTCGTACGTCGTCGACGTGCCGC<br>AGGGTAACACNAAGGAGGAAACNTGCGTGTTCGTCGACACCATTGTCAAATGTAATCTTCATTCCTTAG
PaPYL4.27	ATGTCTCCCAATCGACCCAAATCCTCCCTTCTACTCCAAAGAATCAACGAAACACCCACCACCACCGCCGCCTCCGCCGCGTGCAAACAACAAGCCTACCGCCACCCGACCGCCGCCCATGTCCCCGATCACGT<br>CTCCCGGCACCACAACCACCCCGTCGGCCCCAACCAGTGCTGCTCCGCCGTGTCCCAGCACGTCTCCGCCCCCGTCCCCGCGGTCTGGTCCATCNTCCGCCGCTTC
PaPYL4.28	ATGTCTCCCAATCGACCCAAATCCTCCCTTCTACTNCAAAGAATCAACGAAACACCCACCACCACCGCCGCCTCCGCTGCCGTCGCGTGCAAANATCAAGCCTACCGCCACCCCACCGCCGCCCATGTCCCCGA<br>TCACGTCNCCCGGCACCACAACCACCCCNTCGGCCCCAACCAGTGCTGCTCCGCCGTGTCCCAGCACGTCTCCGCCCCCGTCCCCGCGGTCTGGTCCATCGTCCGCCGNTTCGACAACCCACAGGCGTACAAAC<br>ACTTCGTCAAGACGTGCGACGTCATCCGAGGCGACGGCGACGTCGGCTCGCTCCGCGAGGTCCGCGTCATCTCCGGTCTGCCGGCGGTCAGCAGCACCGAGCGGCTCGAGATCCTCGACGACGAGCGCCACGT<br>CCTCAGCTTCAGCGTCGTCGGAGGGGACCACCGCCTGGCCAACTACCGATCCGTCACCACACTGCACGAGGCACCGACCAACAGCGGAGGCGGGACGGTGGTCGTGGAGTCGTACGTCGTCGACGTGCCGCAG<br>GGTAACACGAAGGAGGAAACNTGCGTGTTCGTCGACACCATTGTCAAATGTAATCTTCATTCCTTAGCTCAGATCTCAGAGAATTTAGCAAGAAGAAACCCCTTCTCCATAA
PaPYL4.29	ATGTCTCCCAATCGACCCAANTCCTCCCTTCTACTCCAAAGAATCAACGAAACACCCACCACCACCGCCGCCTCCGCTGCCGTCGCGTGCAAANATCAAGCCTACCGCCACCCCACCGCCGCCCATGTCCCCGA<br>TCACGTCTCCCGGCACCACAACCACCCCNTCGGNCCCAACCAGTGCTGCTCCGCCGTGTCCCAGCACGTCTCCGCCCCCGTCCCCNCGGTCTGGTCCATCGTCCGCCGCTTCGACAACCCACAGGCGTACAAGC<br>ACTTCGTCAAGACGTGCGACGTCATCCGNGGCGACGGCGACGTCGGCTCGCTCCGCGAGGTCCGCGTCATCTCCGGTCTGCCGGCGGTCAGCAGCACCGAGCGGCTCGAGATCCTCGACGACGAGCGCCACGT<br>CCTCAGCTTCAGCGTCGTCGGNGGGGANCACCGCCTGGCCAACTACCGATCCGTCACCACACTGCACGAGGCNNCGACCAACNGCGGAGGCGGNACGGTGGTCGTGGAGTCGTACGTCGTCGACGTGCCGCA<br>GGGTAACACGAAGGAGGAAACGTGCGTGTTCGTNGACACCATTGTCAAATGTAACCTTCATTCCTTAGCTCAGATCTCAGAGAATTTAGCAAAGAAAGAAACCCTTCTCCATAA
PaPYL4.30	ATGTCTCCCAATCGACCCAAATCCTCCCTTCTACTCCAAAGAATCAACGAAACACCCACCACCACCGCCGCCTCCGCCGCGTGCAAACAACAAGCCTACCGCCACCCGACCGCCGCCCATGTCCCCGATCACGT<br>CTCCCGGCACCACAACCACCCCGTCGGCCCAAACCAGTGCTGCTCCGCCGTGTCCCAGCACGTCTCCGCCCCCGTCCCCGCGGTCTGGTCCATCGTCCGCCGCTTCGACAACCCACAGGCGTACAAGCACTTCG<br>TCAAGACGTGCGACGTCATCCGCGGCGACGGCGACGTCGGCTCGCTCCGCGAGGTCCGCGTCATCTCCGGTCTGCCGGCGGTCAGCAGCACCGAGCGGCTCGAGATCCTCGACGACGAGCGCCACGTCCTCAG<br>CTTCAGCGTCGTCGGAGGGGACCACCGCCTGGCCAACTACCGATCCGTCACCACACTGCACGAGGCACCGACCAACAGCGGAGGCGGGACGGTGGTCGTGGAGTCGTACGTCGTCGACGTGCCGCAGGGTAA<br>CACCAAGGAGGAAACGTGCGTGTTCGTCGACACCATTGTCAAATGTAATCTTCATTCCTTAGCTCAGATCTCGGAGAATTTAGCAAGAAGAAACCCTTCTCCATAA
PaPYL4.31	ATGTCTCCCAATCGACCCAATATCCTCCCTTCTACTCCAAAGAATCAACGAAACACCCACCACCACCGCCGCCTCCGCTGCCGTCGCGTGCAAANATCAAGCCTACCGCCACCCCACCGCCGCCCATGTCCCCG<br>ATCACGTCTCCCGGCACCACAACCACCCCNTCGGCCCCAACCAGTGCTGCTCCGCCGTGTCCCAGCACGTCTCCGCCCCCGTCCCCGCGGTCTGGTCCATCGTCCGCCGNTTCGACAACCCACAGGCGTACAAG<br>CACTTCGTCAAGACGTGCGACGTCATCCGNGGCGACGGCGACGTCGGCTCGCTCCGCGAGGTCCGCGTCATCTCCGGTCTGCCGGCGGTCAGCAGCACCGAGCGGCTCGAGATCCTCGACGACGAGCGCCACG<br>TCCTCAGCTTCAGCGTCGTCGGNGGGGANCANCGCCTGGCCAACTACCGATCCGTCACNACACTGCACGAGGCNNCGACCAACNGCGGAGGCGGNACGGTGGTCGTGGAGTCGTACGTCGTCGACGTGCCGC<br>AGGGTAACACNAAGGAGGAAACNTGCGTGTTCGTCGACACCATTGTCAAATGTAA
PaPYL4.32	ATGTCTCCCAATCGACCCAAATCCTCCCTTCTACTCCAAAGAATCAACGAAACACCCACCACCACCGCCGCCTCCGCTGCCGTCGCGTGCAAATATCAAGCCTACCGCCACCCCACCGCCGCCCATGTCCCCGA<br>TCACGTCTCCCGGCACCACAACCACCCCGTCGGCCCCAACCAGTGCTGCTCCGCCGTGTCCCAGCACGTCTCCGCCCCCGTCCCCGCGGTCTGGTCCATCGTCCGCCGATTCGACAACCCACAGGCGTACAAGC<br>ACTTCGTCAAGACGTGCGACGTCATCCGAGGCGACGGCGACGTCGGCTCGCTCCGGGAGGTCCGCGTCATCTCCGGTCTGCCGGCGGTTAGCAGCACCGAGCGGCTCGAGATCCTCGACGACGAGCGCCACGT<br>CCTCAGCTTCAGCGTCGTCGGCGGGGACCACCGCCTGGCCAACTACCGATCCGTCACCACACTGCACGAGGCACCGACCAACAGCGGAGGCGGGACGGTGGTCGTGGAGTCGTACGTTGTCGACGTGCCGCAG<br>GGTAACACGAAGGAGGAAACGTGCGTGTTCGTCGACACCATTGTCAAATGTAATCTTCATTCCTTAGCTCAGATCTCGGAGAATTTAGCAAGAAGAAACCCTTCTCCATAA
PaPYL4.33	ATGTCTCCCAATCGACCCAAATCCTCCCTTCTACTNCAAAGAATCAACGAAACACCCACCACCACCGCCGCCTCCGCTGCCGTCGCGTGCAAACATCAAGCCTACCGCCACCCCACCGCCGCCCATGTCCCCGA<br>TCACGTCTCCCGGCACCACAACCACCCCNTCGGCCCCAACCAGTGCTGCTCCGCCGTGTCCCAGCACGTCTCCGCCCCCGTCCCCGCGGTCTGGTCCATCGTCCGCCGATTCGACAACCCACAGGCGTACAAAC<br>ACTTCGTCAAGACGTGCGACGTCATCCGAGGCGACGGCGACGTCGGCTCGCTCCGCGAGGTCCGCGTCATCTCCGGTCTGCCGGCGGTCAGCAGCACCGAGCGGCTCGAGATCCTCGACGACGAGCGCCACGT<br>CCTCAGCTTCAGCGTCGTCGGNGGGGANCANCGCCTGGCCAACTACCGATCCGTCACNACACTGCACGAGGCACCGACCAACAGCGGAGGCGGGACGGTGGTCGTGGAGTCGTACGTCGTCGACGTGCCGCA<br>GGGTAACACCAAGGAGGAAACATGCGTGTTCGTCGACACCATTGTCAAATGTAATCTTCATTCCTTAGCTCAGATCTCGGAGAATTTAGCAAGAAGAAACCCTTCTCCATAA

PaPYL4.34	ATGTCTCCCAATCGACCCAAATCCTCCCTTCTACTNCAAAGAATCAACGAAACACCCACCACCACCGCCGCCTCCGCTGCCGTCGCGTGCAAACATCAAGCCTACCGCCACCCCACCGCCGCCCATGTCCCCGATCACGTCTCCCGGCACCACAACCACCCCGTCGGCCCCAACCAGTGCTGCTCCGCCGTGTCCCAGCACGTCTCCGCCCCCGTCCCCGCGGTCTGGTCCATCGTCCGCCGATTCGACAACCCACAGGCGTACAANCACTTCGTCAAGACNTGCGACGTCATCCGAGGCGACGGCGACGTCGGCTCGCTCCGCGAGGTCCGCGTCATCTCCGGTCTGCCGGCGGTCAGCAGCACCGAGCGGCTCGAGATCCTCGACGACGAGCGCCACGTCCTCAGCTTCAGCGTCGTCGGNGGGGANCANCGCCTGGCCAACTACCGATCCGTCACNACACTGCACGAGGCACCGACCAACAGCGGAGGCGGGACGGTGGTCGTGGAGTCGTACGTCGTCGACGTGCCGCAGGGTAACACCAAGGAGGAAACGTGCGTGTTCGTCGACACCATTGTCAAATGTAATCTTCATTCCTTAGCTCAGATCTCAGAGAATTTA
PaPYL4.35	ATGTCTCCCAATCGACCAAAATCCTCCCTTCTACTTCAAAGAATCAACGAAACACCCACCACCACCGCCGCCTCCGCTGCCGTCGCGTGCAAACATCAAGCCTACCGCCACCCCACCGCCGCCCATGTCCCCGATCACGTCTCCCGGCACCACAACCACCCCGTCGGCCCCAACCAGTGCTGCTCCGCCGTGTCCCAGCACGTCTCCGCCCCCGTCCCCGCGGTCTGGTCCATCGTCCGCCGCTTCGACAACCCACAGGCGTACAAGCACTTCGTCAAGACGTGCGACGTCATCCGAGGCGACGGCGACGTCGGCTCGCTCCGGGAGGTCCGCGTCATCTCCGGTCTGCCGGCGGTCAGCAGCACCGAGCGGCTCGAGATCCTCGACGACGAGCGCCACGTCCTCAGCTTCAGCGTCGTCGGAGGGGACCACCGCCTGGCCAACTACCGATCCGTCACCACACTGCACGAGGCACCGACCAACAGCGGAGGCGGGACGGTGGTCGTGGAGTCGTACGTCGTCGACGTGCCGCAGGGTAACACGAAGGAGGAAACGTGCGTGTTCGTCGACACCATTGTCAAATGTAATCTTCATTCCTTAGCTCAGATCTCAGAGAATTTAGCAAGAAGAAACCCTTCTCCATAA
PaPYL5.1	ATGCCTTCCTCTATTCAGCTTCATAGAATCAACTCTATAAATCACAACATTACCGCCGCCGCCTGCCCCGCCTCTCCGGGGGAGGGTCTACACCAGAAACAGGCCAAAACCTGGGTTTTTCCTGTTCCCGACGAGATCCCGGTGCCGGAAAACATCTTGCACCACCACACCCACGCGGTGTCAGCAAATCAGTGTTGCTCGTCGGCGGCACAGACGATCAAAGCGCCGATTGACGCTGTGTGGTCCTTCGTGCGCTGCTTCGATAACCCACAGGCGTACAAGGTGTTCCTGAAGAGCTGCCACATCATCGTCGGCCACGGAGAGGTGGGGACTCTACGGGAGGTGCGCGTGGTCTCTGGCCTTCCGGCTGCTTGCAGCACGGAGAGGCTAGAGATCCTGGACGACGAGGAGCACGTAATGAGCTTCAGCGTGGTCGGCGGCGAACACCGCCTGCACAACTACCGGTCGGTCACCACCCTCCATGAGGCGGCGGTGGGATCGGGCGGTGTGGGAGAGAGCGGGAGGGATAGAACTGTGGTGGTGGAGTCGTACGTGGTGGATGTGCCGCAGGGAAACACGAAAGAGGAGACGTGCGCGTTTGTTGATACGATTGTTAGGTGTAATTTACAGTCGCTTGCACAGATCGTTGAAAAAATTGGCCAAAAATGA
PaPYL5.2	ATGCCTTCCTCTATTCAGCTTCATAGAATCAACTCTATAAATCACAACATTACCGCCGCCGCCTGCCCCGCCTCTCCGGGGGAGGGTCTACACCAGAAACAGGCCAAAACCTGGGTTTTTCCTGTTCCCGACGAGATCCCGGTGCCGGAAAACATCTTGCACCACCACACCCACGCGGTGTCAGCAAATCAGTGTTGCTCGTCGGCGGCACAGACGATCAAAGCGCCGATTGACGCGGTGTGGTCCGTCGTGCGCCGCTTCGATAACCCACAGGCGTACAAGGTGTTCCTGAAGAGCTGTCACATCATCGTCGGCCAGGGAGAGGTGGGGACTCTACGAGAGGTGCGCGTGGTCTCTGGCCTTCCGGCTGCTTGCAGCACGGAGAGGCTAGAGATCCTGGACGACGAGGAGCATGTAATGAGCTTCAGCGTGGTCGGCGGCGAACACCGCCTGCACAACTACCGGTCGGTCACCACCCTCCATGAGGCGGCGGCGGTATCTGGCGGTGTGGGAGAGAGCGGGAGGGATAGAACTGTGGTGGTGGAGTCGTACGTGGTGGATGTGCCGCAGGGAAACACGAAAGAGGAGACGTGCGCGTTTGTTGATACGATTGTTAGGTGTAATTTACAGTCGCTTGCACAGATCGTTGAAAAAATTGGCCAAAAATGA
PaPYL5.3	ATGCCTTTCCTCTATTCAGCTTCATAGAATCAACTCTATCAATCACATCATTACCGCCGCCGCCTGCCCTGCCTCTCCGGGGGCGGGTCTACACCAGAAACACGCCAAAACCTGGGTTTTTCCTGTTCCCGACGAGATCCTGGTGCCGGAAAACATCTCTCACCACCACACCCACGCGGTGTCAGCAAACCAGTGTTGCTCGTCGGCGGCACAGACGATCAAAGCGCCGATTGATGCGGTGTGGTCCGTCGTGCGCCGCTTCGATAACCCACAGGCGTACAAGGTGTTCCTGAAGAGCTGTCACATCATCGTCGGCCAGGGAGAGGTGTGGACTCTACGAGAGGTGAGCGTGGTCTCTGGCCTTCCGGCTGCTTGCAGCACGGAGAGGCTAGAGATCCTGGACGACGAGGAGCACGTAATGAGCTTCAGCGTGGTCGGCGGCGAACACCGCCTGCACAACTACCGGTCGGTCACCACCCTCCATGAGGCGGCGGCGGGATCGGGCGGTGTGGGAGAGAGCGGGAGGGATAGAACTGTGGTGGTGGAGTCGTACGTGGTGGATGTGCCGCAGGGAAACACGAAAGACGAGACGTGCGCGTTTGTTGATACGATTGTTAGGTGTAATTTACAGTCGCTTGCACAGATCGTTGAAAAAATNGGCCAAAAATGA
PaPYL5.4	ATGCCTTCCTCTATTCAGCTTCATAGAATCAACTCTATCAATCACATCATTACCGCCGCCGCCTGCCCTGCCTCTCCGGGGGCGGGTCTACACCAGAAACACGCCAAAACCTGGGTTTTTCCTGTTCCCGACGAGATCCTGGTGCCGGAAAACATCTCTCACCACCACACCCACGCGGTGTCAGCAAACCAGTGTTGCTCGTCGGCGGCACAGACGATCAAAGCGCCGATTGATGCGGTGTGGTCCGTCGTGCGCCGCTTCGATAACCCACAGGCGTACAAGGTGTTCCTGAAGAGCTGTCACATCATCGTCGGCCAGGGAGAGGTGGGGACTCTACGAGAGGTGAGCGTGGTCTCTGGCCTTCCGGCTGCTTGCAGCACGGAGAGGCTAGAGATCCTGGACGACGAGGAGCACGTAATGAGCTTCAGCGTGGTCGGCGGCGAACACCGCCTGCACAACTACCGGTCGGTCACCACCCTCCATGAGGCGGCGGCGGTATCTGGCGGTGTGGGAGAGAGCGGGAGGGATAGAACTGTGGTGGTGGAGTCGTACGTGGTGGATGTGCCGCAGGGAAACACGAAAGACGAGACGTGCGCGTTTGTTGATACGATTGTTAGGTGTAATTTACAGTCGCTTGCACAGATCGTTGAAAAAATTGGCCAAAAATGA
PaPYL5.5	ATGCCTTCCTCTATTCAGCTTCATAGAATCAACTCTATCAATCACATCATTACCGCCGCCGCCTGCCCTGCCTCTCCGGGGGCGGGTCTACACCAGAAACACGCCAAAACCTGGGTTTTTCCTGTTCCCGACGAGATCCCGGTGCCGGAAAACATCTCTCACCACCACACCCACGCGGTGTCAGCAAACCAGTGTTGCTCGTCGGCGGCACAGACGATCAAAGCGCCGATTGACGCGGTGTGGTCCGTCGTGCGCCGCTTCGATAACCCACAGGCGTACAAGGTGTTCCTGAAGAGCTGTCACATCATCGTCGGCCAGGGAGAGGTGGGGACTCTACGAGAGGTGCGCGTGGTCTCTGGCCTTCCGGCTGCTTGCAGCACGGAGAGGCTAGAGATCCTGGACGACGAGGAGCACGTAATGAGCTTCAGCGTGGTCGGCGGCGAACACCGCCTGCACAACTACCGGTCGGTCACCACCCTCCATGAGGCGGCGGCGGTATCTGGCGGTGTGGGAGAGAGCGGGAGGGATAGAACTGTGGTGGTGGAGTCGTACGTGGTGGATGTGCCGCAGGGAAACACGAAAGACGAGACGTGCGCGTTTGTTGATACGATTGTTAGGTGTAATTTACAGTCGCTTGCACAGATCGTTGAAAAAATTGGCCAAAAATGA
PaPYL5.6	ATGCCTTCCTCTATTCAGCTTCATAGAATCAACTCTATAAATCACAACATTACCGCCGCCGCCTGCCCTGCCTCTCCGGGGGCGGGTCTACACCAGAAACACGCCAAAACCTGGGTTTTTCCTGTTCCCGACGAGATCCCGGTGCCGGAAAACATCTCTCACCACCACACCCACGCGGTGTCAGCAAACCAGTGTTGCTCGTCGGCGGCACAGACGATCAAAGCGCCGATTGACGCTGTGTGGTCCTTCGTGCGCTGCTTCGATAACCCACAGGCGTACAAGGTGTTCCTGAAGAGCTGCCACATCATCGTCGGCCACGGAGAGGTGGGGACTCTACGGGAGGTGCGCGTGGTCTCTGGCCTTCCGGCTGCTTGCAGCACGGAGAGGCTAGAGATCCTGGACGACGAGGAGCACGTAATGAGCTTCAGCGTGGTCGGCGGCGAACACCGCCTGCACAACTACCGGTCGGTCACCACCCTCCATGAGGCGGCGGTGGGATCGGGCGGTGTGGGAGAGAGCGGGAGGGATAGAACTGTGGTGGTGGAGTCGTACGTGGTGGATGTGCCGCAGGGAAACACGAAAGACGAGACGTGCGCGTTTGTTGATACGATTGTTAGGTGTAATTTACAGTCGCTTGCACAGATCGTTGAAAAAATTGGCCAAAAATGA
PaPYL5.7	ATGCCTTCCTCTATTCAGCTTCATAGAATCAACTCTATAAATCACATCATTACCGCCGCCGCCTGTCCCGCCTCTCCGGCGGCGGGTCTACACCAGAAACAGGCCAAAACCTGGGTTTTCCTGTTCCCGACGAGATCCCGTTGCCGGAAAACGTCTTGCACCACCACACCCACGCGTTGTCAGCAAACCAGTGTTGCTCGTCGGCGGCACAGACGATCAAAGCGCCGATTGACGCCGTGTGGTCCGTCGTGCGCCGCTTCGATAACCCACAGGCGTACAAGGTGTTCCTGAAGAGCTGTCACATCATCGTCGGCGACGGAGAGGTGGGGACTCTACGAGAGGTGCGCGTGGTCTCTGGCCTTCCGGCTGCTTGCAGCACGGAGAGGCTAGAGATCCTGGACGACGAGGAGCACGTAATGAGCTTCAGCGTGGTCGGCGGCGAACACCGCCTGCACAACTACCGGTCGGTCACCACCCTCCATGAGGCGGCGGCGGGATCGGGCGGTGTGGGAGAGAGCGGGAGGGATAGAACTGTGGTGGTGGAGTCGTACGTGGTGGATGTGCCGCAGGGAAACACGAAAGACGAGACGTGCGCGTTTGTTGATACGATTGTTAGGTGTAATTTACAGTCGCTTGCACAGATCGTTGAAAAAATTGGCCAAAAATGAC
PaPYL5.8	ATGCCTTCCTCTATTCAGCTTCATAGAATCAACTCTATCAATCACATCATTACCGCCGCCGCCTGCCCTGCCTCTCCGGGGTCGGGTCTACACCAGAAACACGCCAAAACCTGGGTTTTTCCTGTTCCCGACGAGATCCCGGTGCCGGAAAACATCTCTCACCACCACACCCACGCGGTGTCAGCAAACCAGTGTTGCTCGTCGGCGGCACAGACGATCAAAGCGCCGATTGACGCGGTGTGGTCCGTCGTGCGCCGCTTCGATAACCCACAGGCGTACAAGGTGTTCCTGAAGAGCTGTCACATCATCGTCGGCCAGGGAGAGGTGGGGACTCTACGAGAGGTGCGCGTGGTCTCTGGCCTTCCGGCTGCTTGCAGCACGGAGAGGCTAGAGATCCTGGACGACGAGGAGCACGTAATGAGCTTCAGCGTGGTCGGCGGCGAACACCGCCTGCACAACTACCGGTCGGTCACCACCCTCCATGAGGCGGCGGCGGTATCTGGCGGTGTGGGAGAGAGCGGGAGGGATAGAACTGTGGTGGTGGAGTCGTACGTGGTGGATGTGCCGCAGGGAAACACGAAAGACGAGACGTGCGCGTTTGTTGATACGATTGTTAGGTGTAATTTACAGTCGCTTGCACAGATCGTTGAAAAAATNGGCCAAAAATGA
PaPYL5.9	ATGCCTTCCTCTATTCAGCTTCATAGAATCAACTCTATAAATCACAACATTACCGCCGCCGCCTGCCCCGCCTCTCCGGGGGAGGGTCTACACCAGAAACAGGCCAAAACCTGGGTTTTTCCTGTTCCCGACGAGATCCCGGTGCCGGAAAACATCTTGCACCACCACACCCACGCGGTGTCAGCAAATCAGTGTTGCTCGTCGGCGGCACAGACGATCAAAGCGCCGATTGACGCTGTGTGGTCCTTCGTGCGCTGCTTCGATAACCCACAGGCGTACAAGGTGTTCCTGAAGAGCTGCCACATCATCGTCGGCCACGGAGAGGTGGGGACTCTACGAGAGGTGCGCGTGGTCTCTGGCCTTCCGGCTGCTTGCAGCACGGAGAGGCTAGAGATCCTGGACGACGAGGAGCACGTAATGAGCTTCAGCGTGGTCGGCGGCGAACACCGCCTGCACAACTACCGGTCGGTCACCACCCTCCATGAGGCGGCGGCGGTATCTGGCGGTGTGGGAGAGAGCGGGAGGGATAGAACTGTGGTGGTGGAGTCGTACGTGGTGGATGTGCCGCAGGGAAACACGAAAGACGAGACGTGCGCGTTTGTTGATACGATTGTTAGGTGTAATTTACAGTCGCTTGCACAGATCGTTGAAAAAATTGGCCAAAAATGA
PaPYL5.10	ATGCCTTCCTCTATTCAGCTTCATAGAATCAACTCTATACAATCACANCATTACCGCCGCCGCCTGCCCNGCCTCTCCGGGGGNGGGTCTACACCAGAAACANGCCAAAACCTGGGTTTTTCCTGTTCCCGACGAGATCCCGGTGCCGGAAAACATCTNNCACCACCACACCCACGCGGTGTCAGCAAANCAGTGTTGCTCGTCGGCGGCACAGACGATCAAAGCGCCGATTGA
PaPYL5.11	ATGCCTTCCTCTATTCAGCTTCATAGAATCAACTCTATCAATCACATCATTACCGCCGCCGCCTGCCCTGCCTCTCCGGGGGCGGGTCTACACCAGAAACACGCCAAAACCTGGGTTTTTCCTGTTCCCGACGAGATCCCGGTGCCGGAAAACATCTCTCACCACCACACCCACGCGGTGTCAGCAAACCAGTGTTGCTCGTCGGCGGCACAGACGATCAAAGCGCCGATTGACGCGGTGTGGTCCGTCGTGCGCCGCTTCGATAACCCACAGGCGTACAAGGTGTTCCTGAAGAGCTGTCACATCATCGTCGGCCAGGGAGAGGTGGGGACTCTACGAGAGGTGCGCGTGGTCTCTGGCCTTCCGGCTGCTTGCAGCACGGAGAGGCTAGAGATCCTGGACGACGAGGAGCANGTAATGAGCTTCAGCGTGGTCGGCGGCGAACACCGCCTGCACAACTACCGGTCGGTCACCACCCTCCATGAGGCGGCGGCGGTATCTGGCGGTGTGGGAGAGAGCGGGAGGGATAGAACTGTGGTGGTGGAGTCGTACGTGGTGGATGTGCCGCAGGGAAACACGAAAGACGAGACGTGCGCGTTTGTTGATACGATTGTTAGGTGTAATTTACAGTCGCTTGCACAGATCGTTGAAAAAATTGGCCAAAAAATGA
PaPYL6	ATGGTTCAAAACATCAACGCACCGCTTCCTTTAGTCTGGTCGATCTTACGCCGTTTTGATCAGCCACAGGTGTACAAGGTATTTGTCAGAAAGTGCATGATGCTGGCGGGCAGCGGCGGCGTTGGGAGCGTAAGGGAGGTGGCGCTCGTCTCCGGCTTGCCCGGTAAAATCGGGAAGGAGAGGCTTGACCGGCTTGATGACGATATGCATGTGATGGTGTATACTATGATCGACGGGGATCAGCGGCTGAAAAACTACCAGTCAACCACCACCGTGCATGAGGCGGAGGGCGGCAGCGGGACGGTGGTTGTCGAGTCGTACGTGGTCGATGTGCCGGAGGGGAACAGCGAGGAGGAGACGCGTGTTTTTGTTAATACCGTTTTAGGTTGTAATCTTAGGTCTTTGGCTAGGGTAAGTGAGGCGTTGGCTTTTAATTATAATTTAAACTAA
PaPYL7	ATGGAGGCAGAGTATATACGTAGATACCATAGGCACCAGTTCAACGAGAGCCAGTGTTCTTCTTGTGTTGTGAAGCACATCAAAGCCCCTGTTGATATTGTCTGGTCATTGGTGAGGAGATTTGATGAGCCACAAAGTTACAAGCCCTTTGTTAGTCGATGCACGTTGCGTGGTGGGGATATGAATATTGGGAGTGTTAGAGAGGTTAATGTTAAGTCTGGACTTCCAGCCACAACAAGCACCGAGCGATTGGAAATTCTTGACGACCAGAAACATATTATCGGGGTCAAATTTGTCGGTGGTGATCACCGTCTAAACAATTACTCATCGATAATCACGGTCCATCCAGATAAAGTAGATGGGACGACAGAAGGGACCATAGTTATCGAGTCATTTGTGGTGGACGTGCCTCAAGGAAATACACTAGACGAGACTTGTTACTTTGTTAAAGCTCTGATAAACTGCAACCTCAAGTCCTTGGCTGACGTCTCCGAACGAATGGCAATCAATGCAAGCTCGGTTTGA
PaPYL8	ATGGACGTCAAAGGACTGAATGGATTGGAGAAAGAGTATATAAAGAAGCATCACAAGCACGAGATTAAGGATAACCAATGCAATTCATTCCTCATCAAGCATATTAAAGCGCCTGTTCATCTCGTTTGGTCTTTGGTCAGGAGGTTCGATCAACCACAGAACTACAAGCCTTTTGTGAGCCGTTGTGTTGTGCAGGGAAATCTTGAAGTCGGCTGTCTAAGGGAAGTTGATGTCAAGTCGGGTCTTCCTGCTACGACAAGCACTGAGAGATTAGAGCTTCTTGATGATAATGAACGCATAATCATCGTCAGTTTTATCGGTGGGGACCACAGGCTCAAAAACTACTCTTCCATTGTGTCTGTCCATCCCGAGATCATCGAAGGGAGACCTGGGACCATGGTAATCGAGTCATTTGTGGTCGATATTCCTGAAGGAAACACAAAAGACGAGACATGCTACTTTGTCGAATCATTAATCAATTGCAATCTTAAATCGCTTGCGAATGTTTCGGAAAAGCTTGCACTGCAAGACACAGCTGAATCAATCGATCGTGTCTAA

PaABIL1	ATGGAAGAGATTTCTCCAGTTACACTTGGCTTAAATAACCATCTGTCTGTTAATCCGGCAATTACGAATCCCTTAGAAATTACACTACTCAAATTAGAAGTGGAAACGGCAAGCCTTCTATCGGACCCAGCTAT<br>CATAATAAACGAGATGGTGGAAAATAAAAGAAAACCCGATATTTTAGTTCAAAATCTGCAAGAAAGTGAAGAAAATGAAGTTATCTCAGTCATGGAAGACCCTACTTTATTGACAAGTGTCGGTTTACTACCA<br>CCCTTAGATGGTATTTCAAATTTAGATGTGCCTTCAGATACTTCTCTAGAGACCGGCTTACCGATAGCTGTCGAGATTGAGGGCACTGGAAGTGGTCAAATACTTGCTAAGGTCATTAGTTTGGAGGAGAGAAG<br>TATAAGACGGAAGTTGTCTGACGAAATCGCCACAGTAGCTGACAAACAAAATCAAGAATGTTCGAGTGGGCCCACTATAAAGGAAACGGTGGTTGCTGTTAAGTTATCGAGTGAAGGGGATTCTAATAAAGGC<br>GGTGTTAAGAGTGTTTTTGAATTGGACTGTAAGCCTCTTTGGGGTTCGGTTTCTGTTCGTGGGCATAGGGCGGAAATGGAAGATGCGGTTATGGCTGTTGCCAATTTCATGAAAATTCCTATTAAGATGTTTGTT<br>GGCGATAATGGAGTAGATGGGATAAGTCGGACTTTGAGTCACCTGACGTCGCATTTTTTCGGGGTTTACGATGGTCATGGTGGATCTCAGGTTGCGAATTATTGCCGTGATCGTCTCCATTTTGCCCTGGAAGAG<br>GAGTTAAAAAACGTTAAAGACATTCTCGTCGATGGGAGTATAAGAAACACTCGGCAGGTCCAATGGGAAAATGTCTTCACCAGTTGCTTTTTAAAAATCGACAATGAAGTTGGAGGAAAGGTGACTCAACTTG<br>ACCGTAAAGGAGATAGTAGTACAGGCATGAGCCCGTCTGTACCGATCACCCCGGAAACAGTGGGGTCCACTGCAGTTGTTGCTGTTGTTTGTTCGTCTCATATTATAGTTGCGAATTGTGGTGATTCAAGGGCA<br>GTACTTTATCGTGGTAAAGAAGCTATCCCGTTATCTATTGATCATAAACCGAATCGTGAGGATGAATATGCTAGGATTGAAGCGTCTGGTGGAAAGGTCATACAGTGGAATGGGCACCGTGTTTTTGGTGTTCT<br>TGCGATGTCACGGTCTATTGGTGATAGATACTTGAAGCCATGGATTATCCCGAAACCAGAAGTGATGTGTGTGCCTCGTGCTCGGGAGGATGATTGCCTTGTTCTAGCAAGCGATGGGTTATGGGATGTGATGT<br>CTAACGAGGAGGTATGCGAGTTGGCACGAAAACGTATTTTAATTTGGCACAAAAAGAACGGAACAACTAATCCGTTAGCGGATAGGGGTGGAGGGGTGGACCCGGCTGCACAAGCAGCAGCCGAGTACCTCT<br>TGCACGTGGCCCTACAGAAAGGGAGCAAGGATAATATTTCCATAATCGTAGTGGATTTGAAAGCTCAAAGAAAGTTTAAGAGTAAATCCTGA
PaABIL2	ATGTCTATTCAGTCGGGGAAGGTGACGGTGAGTGGAAAGACGGGCCGTAGTATTTTCGAGGTGGATTGTGTGCCGTTATGGGGTTTTACGTCCGTGTGCGGTAGGAGGCCGGAGATGGAGGATGCCGTCGCGA<br>CAGTGCCGAGGTTGTTGAACATCCCGATTAGGATGTTGATCGGTGATCGTCGGGTCGATGGATCGGGCTCGTGTTTGAGTCATTTAAGTGGACATTTTTTCGGAGTTTACGATGGTCACGGAGGTTCGCAGGTTG<br>CTAATTATTGTCGAGATCGTCTTCATCGTGCTTTGTCGGAGGAGGTGGAAACTATCATAGCGGACAACACGAACGACGGAAGTAAATGCGAAGAGCATTGGAAAAGAGCTTTCATGAAATGTTTTATAAAAGT<br>GGACGATGAAATCGGAGATAAATCAAGAATCGAACCCATCGCACCGGAAACCGTTGGCTCGACTGCAGTTGTTGCCGTGGTTTGTTCTTCGCATATAATCGTGGCTAATTGTGGGGATTCGAGGGCTGTGCTAT<br>GCCGTGGCAAAGAACCCGTGCCCCTATCCGTGGACCATAAGCCGAGTCGGGAAGATGAATATGCAAGAATCGAAGCGGCCGGAGGTAAAGTGATACAGTGGAACGGACATCGTGTTTTCGGGGTCCTTGCAA<br>TGTCTAGATCCATTGGCGATAAATATTTGAAACCTTGGATAATTCCGGATCCCGAAGTGACTTTCGCAGCTCGAGCCAAGGAAGACGAGTGCCTTGTTCTAGCGAGCGATGGTTTATGGGATGTCATGACGAAC<br>GAGGAGGTTTGTGAAATTGCCCGTAAACGAATACTACTCTGGCATAAAAATAACGGGCCCACGCTCCCTTTAGAAAGGGGTCGAGGGATTGATCCCGCGGCTCAAGCCGCGGCCGAGTACTTGTCAAATCGCG<br>CGCTTCAAAAGGGCAGCAAGGATAACATCACTGTAGTTGTCGTCGACTTGAAGGCTCAGAGAAAGATAAAGAGCAAAAAAATAGAATAA
PaPP2CAL1	ATGGGTTATCTGAATTCTGTGTTGTCCCAATCTTCGAGCAAGGTCCACGTGGATGATGCTCCGGTCAGCGGCGGCGGCCTTAGTCAGAATGGAAAGTTCAGCTATGGATATGCAAGTTCTCCAGGCAAGAGGTC<br>TTCAATGGAGGATTTTTACGAGACAAGGATTGATGGTGTAGATGGAGAGGTCGTTGGTCTCTTTGGCGTCTTTGATGGTCATGGGGGAGCTCGAGCTGCAGAATATGTTAAACACAACCTTTTCAGTAATCTGA<br>TCAGGCACCCAAAGTTTATTTCTGACACCAAATCAGCCATCGCCGATGCATATAGTCATACCGACTCAGAATTTCTGAAATCGGAAAACAATCAGAACCGAGATGCTGGGTCAACTGCTTCTACTGCTATCCTT<br>GTTGGCGACCGTTTATTGGTGGCAAATGTTGGGGACTCTAGGGCTGTTATTTGCAGGGGTGGTAATGCTATTGCTGTCTCTCGAGATCACAAACCGGATCAGACAGACGAGAGGCAAAGAATTGAAGATGCTG<br>GAGGTTTTGTCATGTGGGCAGGAACTTGGAGAGTCGGAGGAGTGCTTGCTGTATCTCGTGCTTTTGGTGATAGACTTCTGAAGCAGTATGTGGTTGCTGATCCTGAGATTCAGGAGGAAAAAGTTGATGACACC<br>CTCGAGTTTCTTATCCTTGCAAGTGATGGACTTTGGGATGTGGTTACAAATGAGGAAGCGGTGTCTATGACTAAGCCAATATCGGATCCAGAAGAAGCGGCGAAGAGGCTAATGCAGGAAGCTCATCAGAGGG<br>GCAGTGGTGACAATATCACTGTTGTCGTTGTTCGGTTTCTTGCCAACCCAGAGGGGTCCTCATACTAA
PaPP2CAL2	ATGTTATGCAACAGTATTTTGAGGTCTGTAAATTATTGGGCGGGACATATTGGACGATACACCGACTTCAGCCAGTTTAGCTATAAAGGTGGAGGTTTGAATGCCACTCAAAGCTTAAATTATTTGTGGAGTCG<br>TAAGTTTGGAAGAACGAGAAAGATGATGGTGGATTCTGGTGCTGCTGCTGAAGGATCTGATATTTTGTTACCTGAGAAGTATGATGATGGTAGCTATGCTAGCGGAGGATGGAAGAGTGAGGATGAAACATTA<br>AGTTGTGGATATTCCACTTTCAGAGGAAAGAGGGCTAGCATGGAGGACTTCTATGACATTAAGGCTTCCAAGATTGACGGACAGCCTGTCTGCCTTTTCGGCATATTTGATGGTCATGGTGGCGCTCGTGCGGC<br>TGAATTTTTGAAGGAACATCTCTTTGAGAATCTCCTGAAGCATCCAGGATTTATTGCAGATACCAAATTGGCTATAAGTGAAACATATCAACAGACTGACCGAGACTTTTTGGAGTCTGAAAAGGAAACCTTTA<br>GAGATGATGGCTCAACAGCTTCGACAGCAGTTCTTGTAGGCAATCACCTTTATGTTGCTAATGTTGGCGATTCTCGAACTATTATATCAAAGGCTGGGAAAGCAATTCCACTCTCAGAAGATCACAAACCAAAT<br>AGAAGTGATGAGAGAAAGAGAATTGAAAGTGCTGGAGGTATTATAATGTGGGCAGGTACTTGGAGGGTTGGTGGGGTATTGGCCATGTCTCGTGCCTTCGGAAATCGCATGCTTAAACAATTTGTTGTGGCTG<br>AACCTGAGATTCAGGATCTGGAGGTGGACCAAGACTTTGAATTGCTTGTGCTTGCCAGTGATGGGCTTTGGGATGTTGTGCCTAATGAGGATGCTGTTTCAATAGCACAAAGTGAAGATGATCCAGAAGGAGCT<br>GCTCGCAAACTGACTGAGACAGCTTTCACTCGTGGCAGTGCTGACAATATCACATGCATTGTCGTCAAGTTCCGCCATGCAAAACCGAATTCCGAGGATACCCATCAAAATCTTGAACCCAAACTCCAACCAAA<br>TGAAACCCAGCAGAATATTATCACATCTAATTTCGAGCCCCCGTCCCTTGAAACTCGTGAAACTTCAAAAGTGGAGCTCGAGGAAACCCTGGAATCTTCTAATGCAGAGCNGGAAGAAAAAAATGTTCAAAGT<br>TGA
PaPP2CAL3	ATGAGACCGGAAGTTAGAAACGACAAAAGATTAAACGATCGGTCGTTGATGTGCCGCGTCAGGCGATCTAGATACGGGACGATGACTCATCTCGGCCGGCGACGAGAAATGGAGGACGCGGCGGCGGTCGAG<br>TTAGGTTTCCTGAGCAAAGGTGGCAAGCGTTACGATTTTTACGGCGTGTACGACGGCCATGGCGGGTGGCGCGTTGCCAAAACCTGCAGCGAGATGATGCACAGGTTGTTGGAGAAGGTTCTGGAAGATGAGA<br>GCGGGGTGGAAATAGGGTGGGGGAAGGTGATGGCGTCGGCGTTTAAGGATATGGATGAGGAGGTGAATAAAAGTGGCGCTGACGTGGCGACGACGAGGGCGGTGATCTGCAGAGGTGGTGTTGCCGTACAGT<br>TGTCCGACGATCACAAGCCTGATAGACCTGATGAACTCGAGAGAATCGAAGTCTGCGGAGGGAAAGTCATTAACTGGAATGGGGCCAGAGTCTTAGGGGTTCTTGCTACCTCGAGATCTATAGGTGATCGTTA<br>CCTGAAACCATACGTGATCGCTGACCCGGAAGTGAAAATAATCAATAGAAGTAGCACAGACGAGTTCTTGATACTGGCGAGTGATGGGCTATGGGATGTCGTCTCAAATGAACTCGCATGCAAGGTTACAAGG<br>AGGTGTTTGGATGGTCGTATGACAAGAAGTTCTCCCTATTCAAGAATAACGAACAAAGATGATATTGAAGGGAGTCGTGATGACGAGTTTGATTTTAAAACGAGTGTTGAAAATCCTCGTTGTTTTGAGGCGGC<br>TGCTGTACTGACACAGTTGGCGATGGCTAGAGGCAGCAATGATAATGTAAGTGTCATTGTGGTGGACATGAGTTAA
PaPYL4 +0 FW	ATGAAAATGTCTCCCAATCGA
PaPYL4 +738 REV	AGAAATTTATGGAGAAGGGTTTC
PaPYL4 pET FW	AAAAAACAATTGATGAAACCATGTCTCCCAATCGA
PaPYL4 pET REV	AAAAAAGAGCTCAGAAATTTATGGAGAAGGGTTTC
PaPYL4 pBD Gibsson FW	TTGACTGTATCGCCGGAAATGAAAATGTCTCCCAATCGA
PaPYL4 pBD Gibsson REV	GACTCACTATAGGGCTCTAGAGAGAAATTTATGGAGAAGGGTTTC
PaPYL4 pGE Gibsson FW	GGCGACCATCCTCCAAAATGGATGAAAATGTCTCCCAATCGA
PaPYL4 pGE Gibsson REV	GAGTCGACCCGGGAATTCAGAAATTTATGGAGAAGGGTTTC
PaPYL4 pHeGHPB FW	AAAAAAGAATTCATGAAAATGTCTCCCAATCGACCCA
PaPYL4 pHeGHPB REV	AAAAAACCCGGGTGGAGAAGGGTTTCTTCTTGCTAAATT
PaPYL6 +0 FW	ATGGTTCAAAACATCAACGC
PaPYL6 +474 REV	GTTTAAATTATAATTAAAAGCCAACGCCT
PaPYL6pBD Gibsson FW	AGTTGACTGTATCGCCGGAAATGGTTCAAAACATCAACGC
PaPYL6 pBD Gibsson REV	GACTCACTATAGGGCTCTAGAGGTTTAAATTATAATTAAAAGCCAACGCCT
PaPYL6 pGE Gibsson FW	GGCGACCATCCTCCAAAATGGATGGTTCAAAACATCAACGC
PaPYL6 pGE Gibsson REV	GAGTCGACCCGGGAATTCTTAGTTTAAATTATAATTAAAAGCCAACGCCT
PaPYL7 +0 FW	ATGGAGGCAGAGTATATACGTA
PaPYL7 +651 REV	TCAAACCGAGCTTGCATTG

PaPYL7 pBD Gibsson FW	AGTTGACTGTATCGCCGGAAATGGAGGCAGAGTATATACGTA
PaPYL7 pBD Gibsson REV	GACTCACTATAGGGCTCTAGAGTCAAACCGAGCTTGCATTG
PaPYL7 pGE Gibsson FW	GGCGACCATCCTCCAAAATCCATGGAGGCAGAGTATATACGTA
PaPYL7 pGE Gibsson REV	GAGTCGACCCGGGAATTCTCAAACCGAGCTTGCATTG
PaPYL8 +0 FW	ATGGACGTCAAAGGACTGAATG
PaPYL8 +681 REV	TTAGACACGATCGATTGATTCAGC
PaPYL8 pBD Gibsson FW	AGTTGACTGTATCGCCGGAAATGGACGTCAAAGGACTGAATG
PaPYL8 pBD Gibsson REV	GACTCACTATAGGGCTCTAGAGTTAGACACGATCGATTGATTCAGC
PaPYL8 pGE Gibsson FW	GGCGACCATCCTCCAAAATGGATGGACGTCAAAGGACTGAATG
PaPYL8 pGE Gibsson REV	GAGTCGACCCGGGAATTCTTATTAGACACGATCGATTGATTCAGC
PaPYL5 +0 FW	ATGCCTTCCTCTATTCAGC
PaPYL5 +730 REV	TCAAACAATATACACATTATCTCTGA
PaPYL5 pBD Gibsson FW	AGTTGACTGTATCGCCGGAAATGCCTTCCTCTATTCAGC
PaPYL5 pBD Gibsson REV	TCAGAGATAATGTGTATATTGTTTGACTCTAGAGCCCTATAGTGAGTC
PaPYL5 pGE Gibsson FW	GGCGACCATCCTCCAAAATGGATGCCTTCCTCTATTCAGC
PaPYL5 pGE Gibsson REV	GAGTCGACCCGGGAATTCTCATTTTTGGCCAATTTTTTCA
PaPYL5 pHeGHB FW	AAAAAAGAATTCATGCCTTCCTCTATTCAGC
PaPYL5 pHeGHB REV	AAAAAACCCGGGTCAAACAATATACACATTATCTCTGA
PaABIL1 +0 FW	ATGGAAGAGATTTCTCCAGTTACA
PaABIL1 +1563 REV	TCAGGATTTACTCTTAAACTTTCTTT
PaABIL1 pACT FW	AAAAAACCCGGGTATGGAAGAGATTTCTCCAGTTACA
PaABIL1 pACT REV	AAAAAACAATTGTCAGGATTTACTCTTAAACTTTCTTT
PaABIL1 pGE Gibsson FW	GGCGACCATCCTCCAAAATCCATGGAAGAGATTTCTCCAGTTACA
PaABIL1 pGE Gibsson REV	GAGTCGACCCGGGAATTCTCAGGATTTACTCTTAAACTTTCTTT
PaABIL2 +0 FW	ATGGGTTATCTGAATTCTGTGTTGT
PaABIL2 +870 REV	TTAGTATGAGGACCCCTCTGG
PaABIL2 pACT FW	AAAAAACCCGGGTATGGGTTATCTGAATTCTGTGTTGT
PaABIL2 pACT REV	AAAAAACAATTGTTAGTATGAGGACCCCTCTGG
PaPP2CAL1 +0 FW	ATGTTATGCAACAGTATTTTGAGGT
PaPP2CAL1 +1206 REV	TCAACTTTGAACATTTTTTTCTTCC
PaPP2CAL1 pACT FW	AAAAAACCCGGGTATGTTATGCAACAGTATTTTGAGGT
PaPP2CAL1 pACT REV	AAAAAACAATTGTCAACTTTGAACATTTTTTTCTTCC
PaPP2CAL2 +0 FW	ATGAGACCGGAAGTTAGAAAC
PaPP2CAL2 +939 REV	TTAACTCATGTCCACCACAATGA
PaPP2CAL2 pACT FW	AAAAAACCCGGGTATGAGACCGGAAGTTAGAAAC

PaPP2CAL2 pACT REV	AAAAAACAATTGTTAACTCATGTCCACCACAATGA
PaPP2CAL3 +0 FW	ATGTCTATTCAGTCGGGGAAGG
PaPP2CAL3 +1041 REV	TAAAAGAGTCGAACGTGTAGT
PaPP2CAL3 pACT FW	AAAAAAGAATTCTCATGTCTATTCAGTCGGGGAAGG
PaPP2CAL3 pACT REV	AAAAAAGAGCTCACTACACGTTCGACTCTTTTA
pGEX +939 FW	GAATTCCCGGGTCGACTC
pGEX +912 REV	ATTTTGGAGGATGGTCGCC
pACT +5'	CTATCTATTCGATGATGAAG
pACT +3'	ACAGTTGAAGTGAACTTGCG
pBD +5'	GTGCGACATCATCATCGGAAG
pBD +3'	AGTCACTTTAAAATTTGTATACACTT
pGEX +5'	TCCAGCAAGTATATAGCATGGCC
pGEX +3'	CCGGGAGCTGCATGTGTCAGAGG
pHeGHPB +5'	GGATCACTCTCGGCATGGAC
pHeGHPB +3'	GATGATGTTTCGGAGCTGGTTG
pET-28a +5'	AGAAGGAGATATACCATGGGCAG
pET-28a +3'	CTTTGTTAGCAGCCGGATCTCAG
PaPYL4 BamHI+	AAAAAAGGATCCATGAAAATGTCTCCCAATCGACC
PaPYL4 SacI-	AAAAAAGAGCTCTTATGGAGAAGGGTTTCTTCTTGCT
PaPYL5 BamHI+	AAAAAAGGATCCATGCCTTCCTCTATTCAGCTTCA
PaPYL5 SacI-	AAAAAAGAGCTCTCATTTTTGGCCAATTTTTTCAACGA

Table S2. Translated sequences of *P. aegyptiaca* ABA receptors from the PPGP EST database.

Name	Sequence
PaPYL4 OrAe42G B1_21428	MKMSPNRPKSSLLLQRINETPTTTAASAACKQQAYRHPTAAHVPDHVSRHHNHPVGPNQCCSAVSQHVSAPVPAVWSIVRRFDNPQAYKHFVKTCDVIRGHGDVGSLREVRVISGLPAVSSTERLEILDEERHVLSFSVVGG DHRLANYRSVTTLHEAPTNSGGGTVVVES YVVDVPQGNTKEETCVFVDTIVKCNLHSLAQISENLARRNPSP
PaPYL5 OrAeBC4_ 4483	MPSSIQLHRINSINHIITAAACPASPGAGLHQKHAKTWVFPVPDEIPVPENISHHHTHAVSANQCCSSAAQTIKAPIDAVWSVVRRFDNPQAYKVFLKSCHIIVGQGEVGTLREVRVVSGLPAACSTERLEILDDEEHVMSFSVV GGEHRLHNYRSVTTLHEAAAGSGGVGESG RDRTVVVESYVVDVPQGNTKDETCAFVDTIVRCNLQSLAQIVEKIGQK
PaPYL6 OrAeBC4_ 485635	MVQNINAPLPLVWSILRRFDQPQVYKVFVRKCMMLAGSGGVGSVREVALVSGLPGRIGKERLDRLDDDMHVMVYTMIDGDQRL KNYQSTTTVHEAEGGSGTVVVESYVVDVPEGNSEEETRVFVNTVLGCNLRSLASVSEALAFNYNLN
PaPYL7 OrAeBC4_ 76947	MEAEYIRRYHRHQLNESQCSSCVVKHIKAPVDIVWSLVRRFDEPQSYKPFVSRCTLRGGDMNIGSVREVNVKSGLPATTSTERLEIL DDQKHIIGVKFVGGDHRLNNYSSIITVHPDKVDGTTEGTIVIESFVVDVPQGNTLDETCYFVKALINCNLKSLADVSERMAINASSV
PaPYL8 OrAe1FB1 _5178	MDVKGLNGLEKEYIKKHHKHEIKDNQCNSFLIKHIKAPVHLVWSLVRRFDQPQNYKPFVSRCVVQGNLEVGCLREVDVKSGLPAT TSTERLELLDDNERIIIVSFIGGDHRLKNYSSVVSVHPEIIEGRPGTMVIESFVVDIPEGNTKDETCYFVESLINCNLKSLANVSEKLAL QDTAESIDRV
PaABIL1 OrAeGnB 1_95861, OrAe41G B1_15188	MEEISPVTLGLNNHLSVNPAITNPLEITLLKLEVETASLLSDPAIIINEMVENKRKPDILVQNLQESEENEVISVMEDPTLLTSVGLLPP LDGISNLNVPSDTSLETGLPIAVEIEGTGSGQILAKVISLEERSIRRKLSDEIATVADKQNQECSSGPTIKETVVAVKLSSEGDSNKGGV KSVFELDCKPLWGSVSVRGHRAEMEDAVMAVANFMKIPIKMFVGDNGVDGISRTLSHLTSHFFGVYDGHGGSQVANYCRDRLHF ALEEELKNVKDILVDGSIRNTRQVQWENVFTSCFLKIDNEVGGKVTQLDRKGDSSTGMSPSVPITPETVGSTAVVAVVCSSHIIVAN CGDSRAVLYRGKEAIPLSIDHKPNREDEYARIEASGGKVIQWNGHRVFGVLAMSRSIGDRYLKPWIIPKPEVMFVPRAREDDCLVL ASDGLWDVMSNEEVCELARKRILIWHKKNGTTNPLAERGGGVDPAAQAAAEYLSHVALQKGSKDNISIIVVDLKAQRKFKSKS
PaABIL2 OrAeBC5_ 10023.1, OrAe61G B1_31368	MSIQSGKVTVSGKTGRSIFEVDCVPLWGFTSVCGRRPEMEDAVATVPRLLNIPIRMLIGDRRVDGSGSCLSHLSGHFFGVYDGHGGS QVANYCRDRLHRALSEEVETIIADNTNDRSKCEEHWKRALAKCFIKVDDEIGDKSRVEPIAPETVGSTAVVAVVCSSHIIVANCGDS RAVLCRGKEPVPLSVDHKPSREDEYARIEAAGGKVIQWNGHRVFGVLAMSRSIGDKYLKPWIIPDPEVTFAARAKEDECLVLASDG LWDVMTNEEVCEIARKRILLWHKNNGPTLPLERGRGIDPAAQAAAEYLSNRALQKGNKDNITVVVVDLKAQRKIKSKKIE

PaPP2CAL1 OrAeGnB1_136489	MGYLNSVLSQSSSKVHVDDAPVSGGGLSQNGKFSYGYASSPGKRSSMEDFYETRIDGVDGEVVGLFGVFDGHGGARAAEYVKHN LFSNLIRHPKFISDTKSAIADAYSHTDSEFLKSENNQNRDAGSTASTAILVGDRLLVANVGDSRAVICRGGNAIAVSRDHKPDQTDE RQRIEDAGGFVMWAGTWRVGGVLAVSRAFGDRLLKQYVVADPEIQEEKVDDTLEFLILASDGLWDVVTNEEAVSMTKPISDPEEA AKRLMQEAHQRGSGDNITVVVVRFLANPEGSSY
PaPP2CAL2 OrAeGnB1_136067	MLCNSILRSVNYWAGHIGRYTDFRQFSYKGGGLNATQSLNYLWSRKFGRTRKMMVDSGAAAEGSDILLPEKYDDGSYASGGWKSEDETLSCGYSTFRGKRASMEDFYDIKASKIDGQPVCLFGIFDGHGGARAAEFLKEHL FENLLKHPGFIADTKLAISETYQQTDRDFLESEKETFRDDGSTASTAVLVGNHLYVANVGDSRTIISKAGKAIPLSEDHKPNRSDERKRIESAGGIIMWAGTWRVGGVLAMSRAFGN RMLKQFVVAEPEIQDLEVDQDFELLVLASDGLWDVVPNEDAVSIAQSEDDPEGAARKLTETAFTRGSADNITCIVVKFRHAKPNSE DTHQNLEPKLQPNETQQNIITSNFEPPSLETRETSKVELEETLESSNAELEEKNVQS
PaPP2CAL3 OrAeBC4_548578, OrAeBC4_1049359, OrAeBC4_64960	MRPEVRNDIRLNDRSSMCRVRGSRYGTMTHLGRRREMEDAAAVELGFLSKGGKRYDFYGVYDGHGGWRVAKTCSEMMHRLLE KVLEDESGVEIGWGKVMASAFKDMDEEVNKSGADVATTGSTAVVAVVGEEEVVVANCGDSRAVICRGGVAVQLSDDHKPDRPD ELERIEVCGGKVINWNGARVLGVLATSRSIGDRYLKPYVIADPEVKIINRSSTDEFLILASDGLWDVVSNELACKVTRRCLDGRMTR SSPYSRITNKDDIEGSRDDEFNFKTSIENPRCFEAAAVLTQLAMARGSNDNVSVIVVDMS
